# Medicinal Chemistry Perspective on Targeting Mono-ADP-Ribosylating PARPs with Small Molecules

**DOI:** 10.1021/acs.jmedchem.2c00281

**Published:** 2022-05-24

**Authors:** Maria Giulia Nizi, Mirko M. Maksimainen, Lari Lehtiö, Oriana Tabarrini

**Affiliations:** † Department of Pharmaceutical Sciences, University of Perugia, 06123 Perugia, Italy; ‡ Faculty of Biochemistry and Molecular Medicine & Biocenter Oulu, University of Oulu, 5400 Oulu, Finland

## Abstract

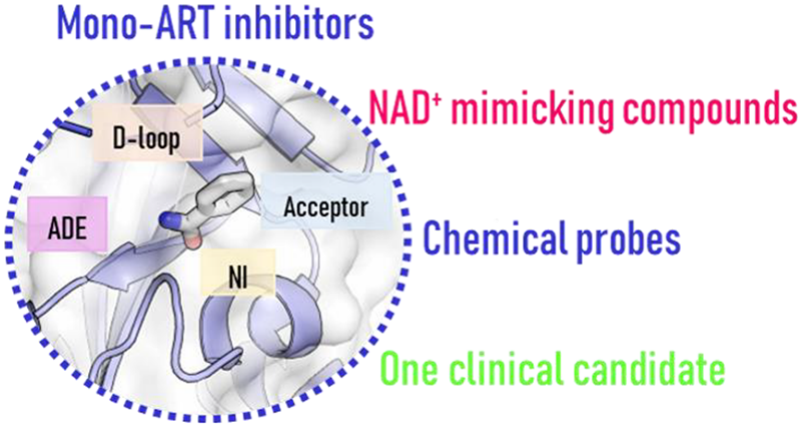

Major advances have recently defined functions for human mono-ADP-ribosylating PARP enzymes (mono-ARTs), also opening up potential applications for targeting them to treat diseases. Structural biology combined with medicinal chemistry has allowed the design of potent small molecule inhibitors which typically bind to the catalytic domain. Most of these inhibitors are at the early stages, but some have already a suitable profile to be used as chemical tools. One compound targeting PARP7 has even progressed to clinical trials. In this review, we collect inhibitors of mono-ARTs with a typical “H–Y−Φ” motif (Φ = hydrophobic residue) and focus on compounds that have been reported as active against one or a restricted number of enzymes. We discuss them from a medicinal chemistry point of view and include an analysis of the available crystal structures, allowing us to craft a pharmacophore model that lays the foundation for obtaining new potent and more specific inhibitors.

## Introduction

ADP-ribosyltransferases catalyze the covalent attachment of ADP-ribose units post-translationally on a variety of amino acid residues of target proteins.^1^ ADP-ribosylation is found in bacteria^2^ as well as in eukaryotes, and indeed, human PARPs, previously referring to “poly-ADP-ribose polymerase”, and tankyrases (TNKS) form a family of diphtheria-toxin-like ADP-ribosyltransferases (ARTDs). The family name comes from the similarities of their catalytic domain with that of diphtheria toxin,^3^ which exerts its pathogenic mechanism by ADP-ribosylating the specific diphthamide residue of the elongation factor 2.^4^ Similarly, many other bacterial toxins specifically label target proteins affecting the host cell functions.^5^ Since the 1960s, when the poly-ADP-ribosylation activity was discovered,^6^ different nomenclatures have been used for these enzymes, as reported in Table 1. In this review, we will use the recent recommended nomenclature^3^ with the term “PARP” (or TNKS) only used in association with the exact number of PARP or TNKS referred to and the terms mono-ARTs and poly-ARTs to indicate the two subfamilies of the mono-ADP-ribosylating (MARylating) and poly-ADP-ribosylating (PARylating) enzymes, respectively.

**Table 1:** Overview of the PARP and TNKS Enzymes of the ARTD Family

activity	protein	other nomenclature	catalytic motif	localization	exemplary functions	noncatalytic domains^a^
poly-ART	PARP1	ARTD1, PARS, ADPRT1	H–Y–E	nuclear/cytosolic	DNA damage repair	3 × ZnF; BRCT; WGR; HD
	PARP2	ARTD2	H–Y–E	nuclear	DNA damage repair	WGR
						HD
	TNKS1	PARP5a; ARTD5	H–Y–E	nuclear/cytosolic	telomere elongation and Wnt/β-catenin signaling	5 × ARC; SAM
	TNKS2	PARP5b; ARTD6	H–Y–E	nuclear/cytosolic	telomere elongation and Wnt/β-catenin signaling	5 × ARC; SAM
mono-ART	PARP3	ARTD3	H–Y–E	nuclear	DNA damage repair	WGR
						HD
	PARP4	vPARP; ARTD4	H–Y–E	nuclear/cytosolic	unknown role in vault particles	BRCT; HD; vWA; MVP BD; VIT
	PARP6	ARTD17	H–Y–I	cytosolic	G2-M cell cycle progression, neurodevelopment	
	PARP7	tiPARP; ARTD14	H–Y–I	nuclear/cytosolic	gene regulation, immune response	ZnF; WWE
	PARP8	ARTD16	H–Y–I	nuclear/cytosolic	cellular apoptosis pathway	
	PARP10	ARTD10	H–Y–I	nuclear/cytosolic	immune response, cell proliferation, DNA damage repair	3 × UIM; 2 × RRM
	PARP11	ARTD11	H–Y–I	cytosolic	immune response, nuclear pore formation	WWE
	PARP12	ARTD12, ZC3HDC1	H–Y–I	cytosolic	immune response, stress granule formation, vesicle trafficking	4 × ZnF; 2 × WWE
	PARP14	BAL2; ARTD8	H–Y–L	nuclear/cytosolic	gene regulation, immune response	RRM; WWE; 3 × MD
	PARP15	BAL3; ARTD7	H–Y–L	nuclear/cytosolic	stress granule formation	2 × MD
	PARP16	ARTD15	H–Y–Y	cytosolic	ER stress response	PRD
inactive	PARP9^b^	BAL1; ARTD9	Q–Y–T	nuclear/cytosolic	DNA damage repair and immune response	2 × MD; DeBD
	PARP13	ARTD13, ZAP, ZC3HAV1	Y–Y–V	cytosolic	immune and stress response	4 × ZnF; WWE

a ZnF: zinc finger. BRCT: BRCA1 carboxy terminal domain. WGR: W, G, R domain. HD: helical regulatory domain. ARC: ankyrin repeat cluster. SAM: sterile alpha motif. vWA: von Willebrand factor type A domain. MVP BD: major vault particle binding domain. VIT: vault protein interalpha-trypsin domain. WWE: W, W, E, domain. UIM: ubiquitin interaction motif. RRM: RNA recognition domain. MD: macrodomain. PRD: putative regulatory domain. DeBD: Deltex binding domain.

b PARP9 complex with an E3 ubiquitin ligase DTX3L is actually able to MARylate ubiquitin.

**Figure 1:**
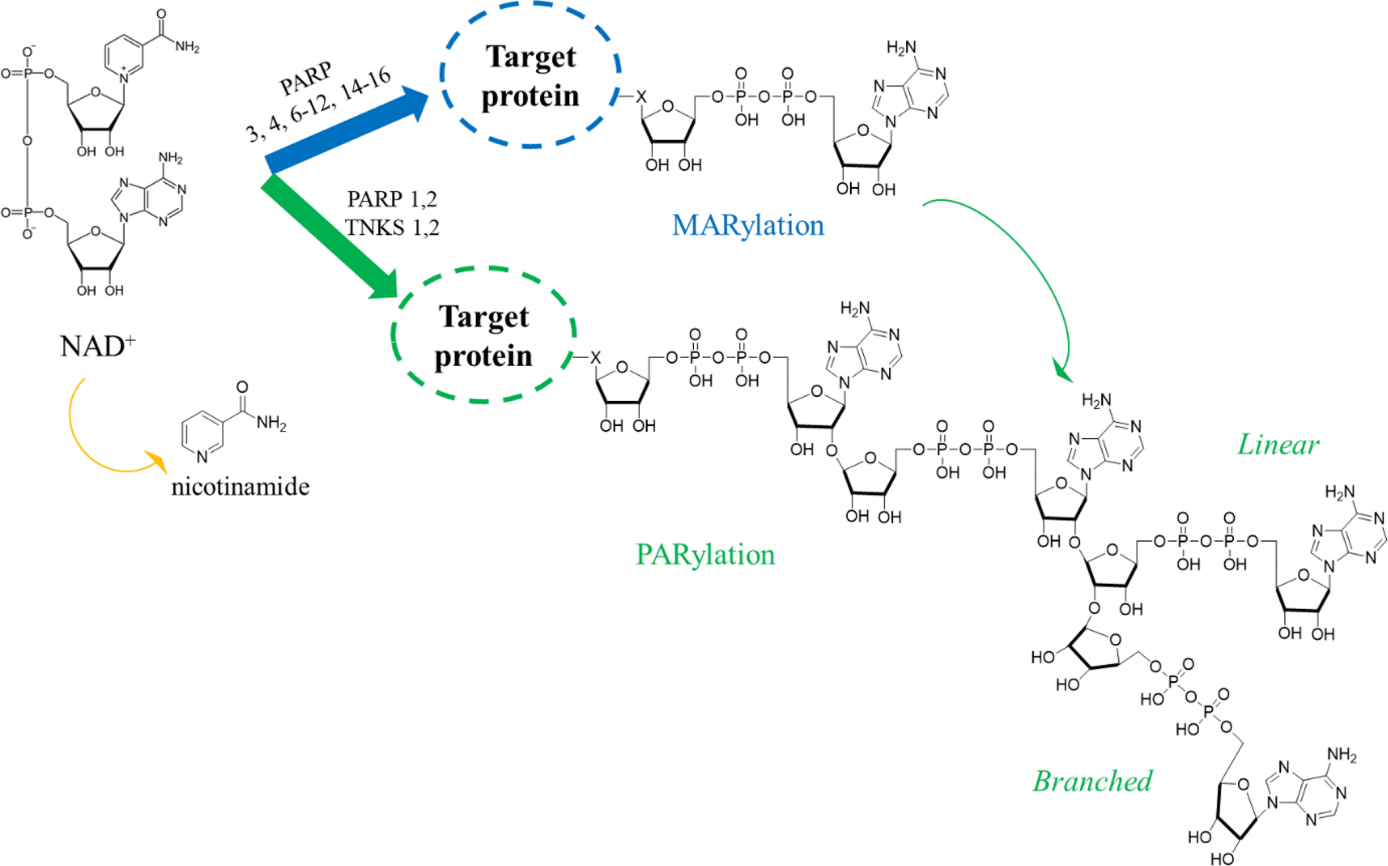
MARylation and PARylation ART mediated as a post-translational modification of proteins with concomitant release of nicotinamide from NAD^+^ as a byproduct. In some cases, MARylated proteins can be also further modified by poly-ARTs PARP1–2 and TNKS1–2 that can elongate the chain.

Different classifications can be used for the various ART subfamilies based on their functions or on the catalytic activity. PARP1, PARP2, and PARP3 can be also defined as DNA-dependent enzymes; TNKS1 and TNKS2 (previously known also as PARP5a and PARP5b) belong to the class of tankyrases; three members contain CCCH zinc fingers (PARP7, PARP12, and PARP13); three enzymes contain two or three macrodomains (PARP9, PARP14, and PARP15).^7^ All of the members share a common catalytic domain, which binds the substrate β-nicotinamide adenine dinucleotide (NAD^+^) and transfers the ADP-ribose units of NAD^+^ to target macromolecules. The nicotinamide portion of the substrate is released as a byproduct (Figure 1). The catalytic domain can be divided into a donor site binding substrate NAD^+^ and an acceptor site where the target macromolecule or the PAR chain to be elongated binds. The donor site is composed of a nicotinamide binding pocket (NI site), a phosphate binding site, and an adenine ribose binding site (ADE site). The NI site is characterized by a highly conserved motif constituted by two tyrosine residues that generate π–π interactions with the nicotinamide ring and serine and glycine residues responsible for two essential hydrogen bonds utilized also by most of the developed pan-PARP inhibitors.^8,9^ The donor site and acceptor site are surrounded by two different loops, a donor loop (D-loop) and an acceptor loop, respectively. The D-loop is not conserved among the subfamilies and influences their catalytic activity. While the catalytic domain is shared by all of the subfamilies, additional noncatalytic domains are found in various PARPs that modulate the enzymatic activity, macromolecular interactions, and protein localization.^10,11^

According to their catalytic activity, ARTs can be classified into mono-ARTs, poly-ARTs, and inactive proteins. Poly-ARTs (PARP1–2 and TNKS1–2) iteratively catalyze the covalent attachment of multiple ADP-ribose units, resulting in poly-ADP-ribosylation (called PARylation for conciseness, Figure 1).

The polymer is formed by α(2–1) *O-*glycosidic bonds, allowing linear (2′–1′′ ribose ribose glycosidic bond) or branched (2″–1″′ ribose ribose glycosidic bond) chains, and can span from a few up to 200 ADP-ribose units.^12^ The PARylation activity mainly results from the presence of a triad of amino acids “H–Y–E” (Table 1) in which histidine and tyrosine are responsible for the correct orientation of NAD^+^, while the glutamate is essential for the elongation of the chain.^13,14^ The glutamate is also involved in stabilizing the oxocarbenium ion transition state of NAD^+^.^13^

The mono-ARTs (PARP3–4, -6–8, -10–12, and -14–16) are not capable of generating polymers but catalyze the addition of a single ADP-ribose unit (MARylation, Table 1, Figure 1). Typically, mono-ARTs present the triad “H–Y−Φ”, in which Φ corresponds to an isoleucine, leucine, or tyrosine residue, thus losing the glutamate critical for the PARylation reaction. In the mono-ART enzymes like PARP10, the oxocarbenium ion is stabilized by a glutamate belonging to the substrate protein, making the modification a substrate-assisted catalysis.^15^ However, the presence of the H–Y–E triad is not a sufficient condition to PARylate the substrate, as demonstrated by PARP3 and PARP4 that, despite the presence of an intact triad, lost the polymerase activity,^16^ suggesting that additional structural features like the donor site lining D-loop could limit the activity of PARP3–4 to MARylation.^14^

The catalytic domains of PARP9 (triad Q–Y–T) and PARP13 (triad Y–Y–V) do not have a histidine that is essential for the binding of NAD^+^, making them inactive (Table 1).^7^ The inactivity of PARP13 has been explained also by a closed and tightly packed active site,^17^ and it should be noted that the PARP9 complex with an E3 ubiquitin ligase is actually able to MARylate ubiquitin.^18^ The C-terminus of DTX3L is capable of doing this reaction alone,^19^ but the role of PARP9 in modulating the activity is unclear.^20^

The lysine, glutamate, aspartate, serine, and cysteine residues of the target proteins have been found to be MARylated and PARylated through the generation of O-, N-, or S-glycosidic bonds. A large amount of proteins has been identified as ADP-ribosylation targets^21−23^ of which some are specific because they are modified by a single enzyme, while others can be a substrate of a variety of enzymes. PARPs are also able to automodify themselves, which could limit their activity and affect their localization and stability.^24^ In addition to proteins, nucleic acids also have been demonstrated to be modified by ADP-ribosylation (reviewed recently by Feijs, Zaja et al.^25^). PARylation adds a large negative charge to the target proteins and subsequently modulates its interactions with other macromolecules but simultaneously acts as a localization signal for, e.g., DNA repair proteins^26^ and tags proteins for ubiquitination and subsequent proteasomal degradation.^27^ MARylation, despite being a smaller modification, causes similar phenomena and can also act as a priming modification for PARylation.^28^

ADP-ribosylation is a reversible process, and several enzymes not only bind the modification but can also hydrolyze it, limiting the signaling event (reviewed recently by Ahel et al.^29^). PAR glucohydrolase (PARG) and ADP-ribosyl hydrolase 3 (ARH3) are able to cleave ribose–ribose bonds and degrade the polymer. PARG cannot remove the proximal serine MARylation prominent in DNA repair, and ARH3 is the enzyme responsible for hydrolyzing this last ADP-ribose unit.^30^ ARH1 can remove the arginine MARylation, while TARG, MacroD1, and MacroD2 hydrolyze the ester bond between the ribose and the acidic amino acids.^31^ Finally, the NUDIX family of proteins is also involved in the degradation by hydrolyzing the phosphodiester bond in the protein-proximal ADP-ribose unit, but they do not remove the modification completely.^32^

DNA-activated PARP1–3 play critical roles in maintaining our genomes. They differ in the various DNA damages they detect and by the resulting modification as PARP1–2 are poly-ARTs, while PARP3 is a mono-ART. The DNA damage recognizing part of the proteins also differs; PARP1 has specialized zinc fingers ZnF1, ZnF2, and ZnF3, the last essential for DNA-dependent activation. PARP2–3 lack these and rely on the WGR domain which is present in all of these proteins. The role of the various domains in PARP1–3 has been recently reviewed by de Oliveira and colleagues.^10^ PARP1 is the main DNA damage sensor and signal transducer and responsible for approximately 90% of PAR chain formation, while PARP2–3 have more specialized roles in the process as reviewed by Lavrik et al.^33^ PARP1–3 are autoinhibited by a helical regulatory domain, which undergoes conformational changes allowing substrate NAD^+^ binding and binding of a histone PARylation factor HPF1.^34,35^ HPF1 forms a joint active site with PARP1–2 and changes the residue specificity of the PARylation to serine, which is the major PARylated residue in DNA repair.^36^ PARylation of PARP itself and of histone tails leads to chromatin remodeling, recruitment of DNA repair factors through their PAR binding module, and release of PARP from the DNA damage site.^37^

**Figure 2:**
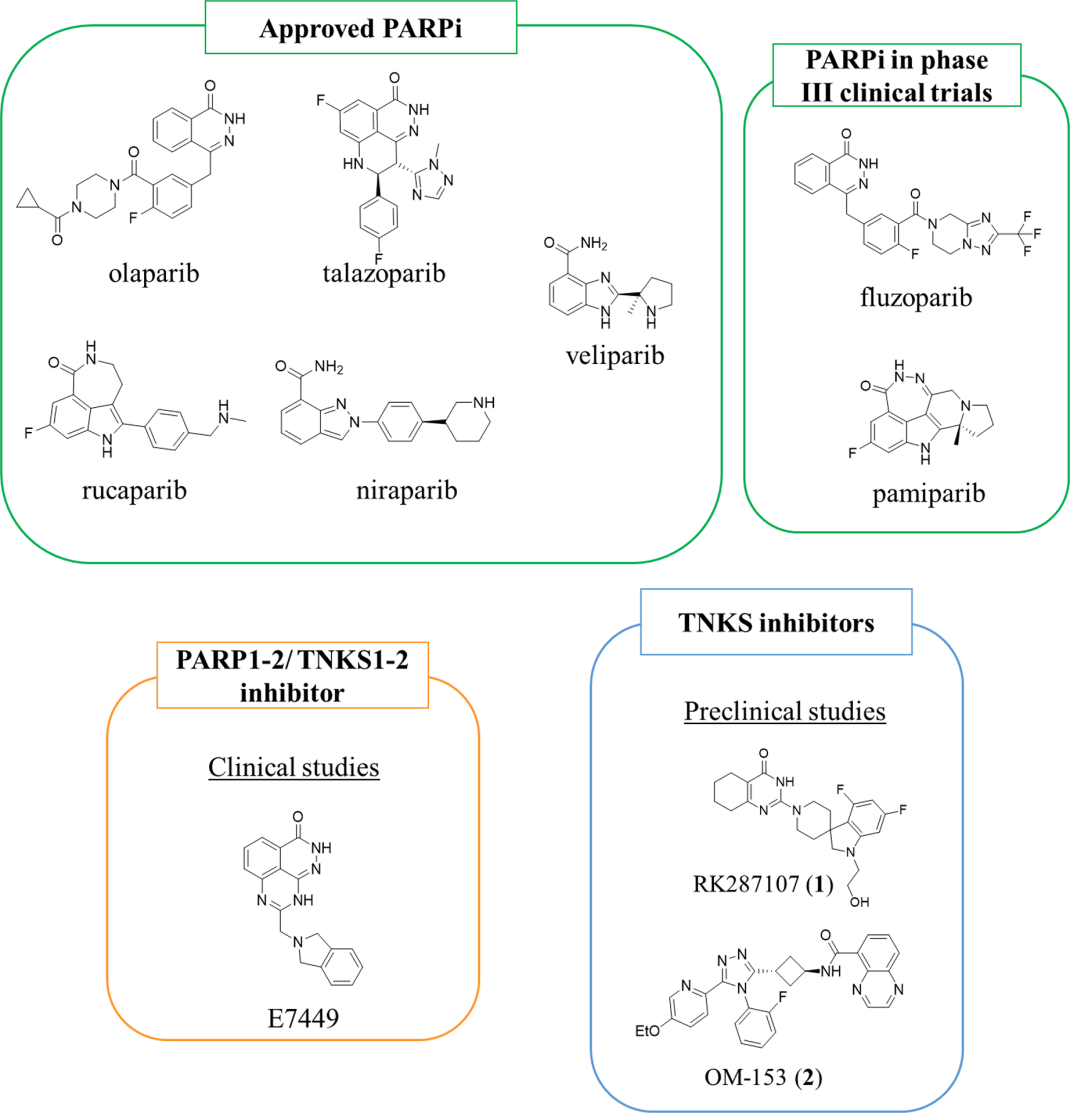
PARPi currently in use as anticancer agents and in phase III of clinical trials, and examples of dual PARP1–2/TNKS1–2 inhibitors in clinical studies and TNKS inhibitors in the preclinical phase.

PARP1–3 are activated by single-strand breaks (SSBs) that are repaired and do not progress to double-strand breaks (DSBs). Inhibition of these PARPs would prevent this repair, and a major advancement in using this property in cancer therapy came when it was discovered that PARP inhibition was synthetically lethal with the BRCA deficiency (breast cancer type 1/2 susceptible).^38,39^ BRCA1/2 are critical enzymes in the resolution of DNA DSBs by promoting the homologous recombination repair (HRR), and as BRCA deficiency is a common feature in multiple cancer cells, including breast and ovarian cancers, PARP inhibition appeared as a magic bullet to target these tumors alone or in combination with DNA damage causing therapy.^40−42^ On the basis of the essential role played by PARP1 in tumor progression and the discovered synthetic lethality, multiple academic and industrial efforts were initiated to improve the early PARP inhibitors (PARPi) toward clinically approved drugs,^43^ and patent literature on new PARPi has expanded^44^ since the PARP1 inhibitor development culminated with the approval of olaparib/Lynparza (AstraZeneca) by the FDA and EMA in 2014 for the treatment of BRCA-deficient ovarian cancers (Figure 2).^45^ This opened the way to a new anticancer treatment that expanded the precision medicine approach, and subsequently, talazoparib^46^ (Pfizer), rucaparib^47^ (Pfizer/Clovis), and niraparib^48^ (Merck/Tesaro) have been approved for the treatment of breast, ovarian, fallopian, peritoneal, pancreatic, and prostate cancers. In addition, veliparib, which is still in phase 3 clinical trials, has been approved for use by the EMA and FDA under an orphan designation for ovarian cancer.^49^ Multiple investigations on the approved drugs and new inhibitors are ongoing to expand the use of PARPi toward a range of other oncologies, including lung cancer and neuroblastoma.^50−55^

Besides the role of PARP1, TNKS1–2 poly-ARTs are currently being investigated as potential therapeutic targets in cancer mainly due to their role in controlling the Wnt/β-catenin signaling pathway. Readers are directed to the following reviews.^56−59^ TNKS1–2 control protein complexes and protein stability through PARylation and targeting proteins to proteasomal degradation. In Wnt/β-catenin signaling they control the so-called “β-catenin destruction complex” consisting of axin, casein kinase 1, adenomatous polyposis coli (APC), glycogen synthase kinase 3 GSK3, and protein phosphatase 2A. TNKSs PARylate axin, which is a limiting component of the complex, leading to reduced phosphorylation of β-catenin. TNKS inhibition thus stabilizes the complex and prevents β-catenin transport to the nucleus. Mutations in the destruction complex proteins, especially in APC, are frequent in cancers, and therefore, TNKS inhibitors could be used as a therapy for treating a range of cancers^60^ including colon,^61^ lung,^62^ liver,^63^ ovarian,^64^ and brain.^65^ Another essential role of these enzymes is explicated in the Hippo signaling. Indeed, they promote the activity of the oncoprotein YAP by suppressing the antagonist angiomotin (AMOT) family proteins involved in the YAP degradation. Elevated expression of YAP has been identified in various human cancers, and as YAP inhibitor development is very limited, TNKS inhibition could be a valid alternative for hampering YAP oncogenic properties.^66^ Several TNKS inhibitors have been developed^67,68^ such as compounds E7449^69^ and STP1002^70^ (structure undisclosed), which have progressed in clinical studies, even if compound E7449 behaves as a dual TNKS1–2 and PARP1–2 inhibitor. Other TNKS inhibitors such as RK287107 (**1**)^71^ and OM-153 (**2**)^72^ are being evaluated in preclinical studies.

While the development of poly-ART inhibitors is widely pursued with new compounds continuing to appear in the literature,^44,67,73−75^ the mono-ART inhibitor development is still in the early stages. However, remarkable advances have been achieved during recent years. The approved PARPi are not selective toward PARP1,^76^ especially the early inhibitors that also inhibit mono-ART enzymes; however, next generation of selective PARP1 inhibitors is emerging.^77^ Also the H–Y–E containing PARP3–4 are typically inhibited by the known PARPi^78,79^ although efforts have been recently made to develop selective inhibitors also for these enzymes.^80,81^ More attention is now paid toward assessing inhibitor selectivity as this is crucial information when using the discovered inhibitors as chemical probes to study the functions of the rather recently discovered and often understudied mono-ARTs.

Many human enzymes with mono-ART activity have been implied to have critical roles in cancer progression and other diseases, and their potential as drug targets is emerging.^82−84^ The first mono-ART inhibitors appeared about 10 years ago, but interest has increased in recent years with many compounds that have been just published, prompting us to collect all of them in this review. In particular, the inhibitors of mono-ARTs “H–Y−Φ” that have been reported as active against one enzyme or restricted toward the mono-ARTs subfamily will be described. We will discuss the identification strategy, the hit-to-lead optimization phase, and the possible SAR studies. When available, the cocrystal structures will be analyzed attempting to highlight the structural features leading to mono-ART selectivity and to derive a pharmacophore model differentiating mono-ART inhibitors from the PARPi described in the earlier literature.

## Mono-ART Inhibitors

Mono-ART inhibitor discovery has often focused on some particular PARP, while efforts have been made to profile the inhibitors also against other enzymes of the family. This has revealed an overlap that can be somewhat expected due to the conserved catalytic domains. Often the discovery has taken advantage of previously described early PARP1 inhibitors, providing nicotinamide mimetic scaffolds that after (structure-guided) optimization led to discover selective/specific mono-ARTs inhibitors. The screening of large compound libraries in some cases permitted one to enrich the scaffold armamentarium. In the following we will address each human mono-ART separately, indicating possible selectivity data that are available for the compounds described in the literature. Many inhibitors have been reported for the best studied PARP10 and PARP14 that will be discussed first. Most of the other mono-ARTs, for which only one paper has been published, will be reported based on structure similarity and biological functions in the following order: PARP11, PARP15, PARP6, PARP12, and PARP16. The review will culminate describing the only clinical candidate, RBN-2397 (**76**), that selectively inhibits the PARP7.

### PARP10 Inhibitors To Modulate Cell Proliferation, Inflammation, and DNA Repair

PARP10 was the first ARTD family member demonstrated to catalyze only mono-ADP-ribosylation.^15^ It is a 150 kDa protein localized both in the cytoplasm and in the nucleus, shuttling between the compartments.^85^ Its interaction partners, e.g., c-Myc and histones, are situated in the nucleus and others such as NEMO in the cytoplasm and some that shuttle between the compartments, similarly to PARP10, like proliferating cell nuclear antigen (PCNA).^86^ Both the catalytic activity as well as the ubiquitin interaction motifs of PARP10 are required for some of the cellular functions of the protein.^87^ PARP10 is able to automodify itself and other proteins on acidic residues. Even if the role of PARP10 is not completely elucidated, it is clear that it is a partner of the proto-oncoprotein c-Myc that functions as a transcriptional regulator involved in cellular apoptosis or proliferation.^88^ The interaction was confirmed by coimmunoprecipitation experiments that highlighted the involvement of the C-terminal half of PARP10 in the c-Myc binding. The PARP10 catalytic domain interacts with a PCNA linking PARP10 to the DNA replication progression under cellular stress conditions.^86^ In particular, upon replication fork arrest, the monoubiquitination of PCNA at its Lys164 generates a cascade of events, essential to restart the stalled fork. A downregulation of PARP10 is responsible for a reduction of the PCNA ubiquitination, thus inhibiting the replication.^89^ Multiple potential protein targets for PARP10 were identified through protein assays.^90^ Validated ones include GSK3β, an enzyme known for its involvement in Wnt signaling, metabolism, immunity, apoptosis, and tumorigenesis.^90,91^ PARP10 is differently expressed in the various tissues with enhanced expression in the thymus and spleen or adipose tissue and liver, suggesting potential roles in innate immunity and metabolism, respectively. On the other hand, PARP10 overexpression in various cancer cell lines along with the known PARP10 target proteins led to the hypothesis that PARP10 promotes cancer proliferation and acts as an oncogene.^92^

**Figure 3:**
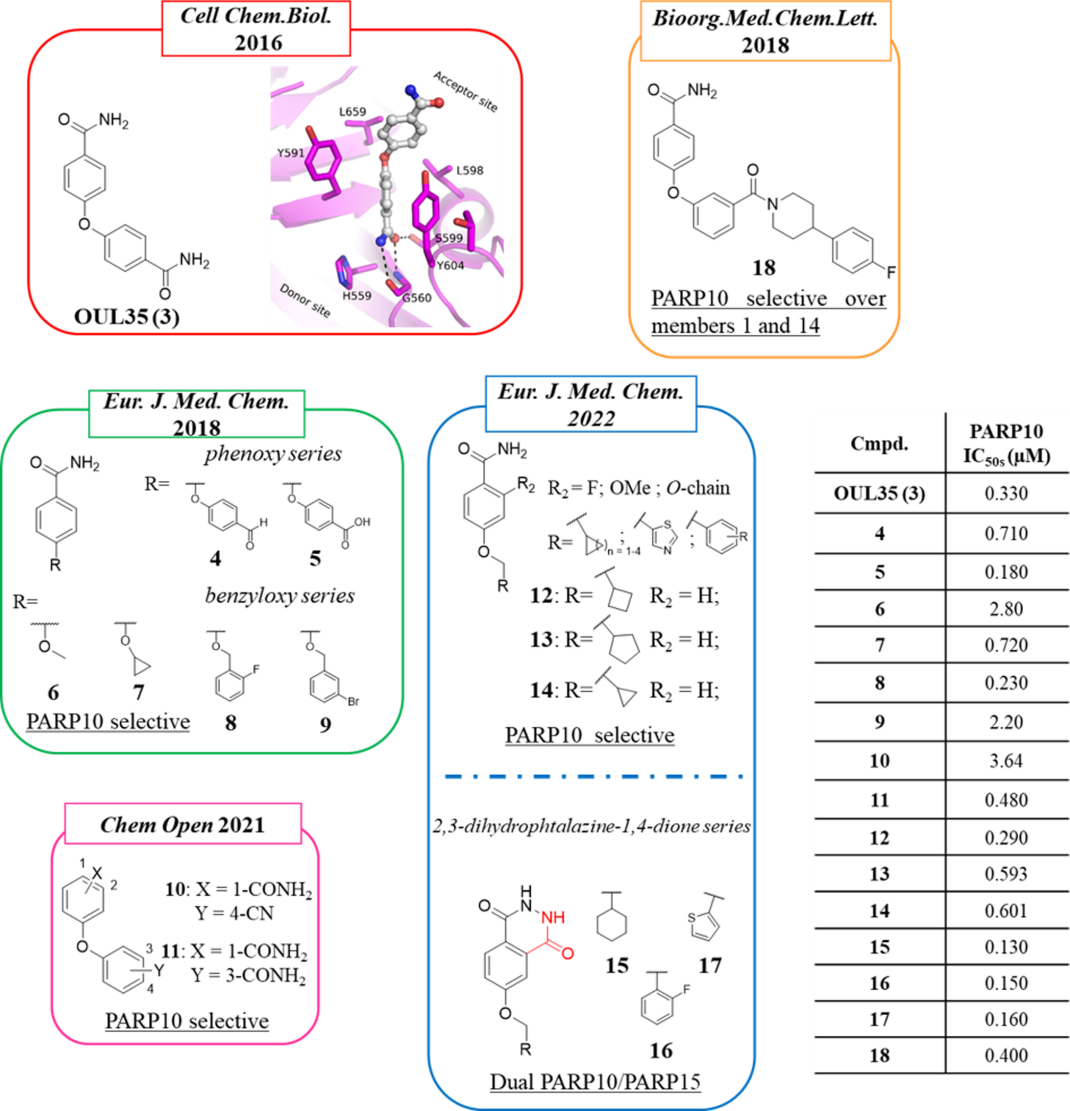
Structure of OUL35 (**3**)^93^ along with its crystal structure with PARP15 gain-of-binding mutant^94^ and its analogues developed by Lehtiö et al.^94^ (in the green box), Schuler et al.^95^ (in the orange box), Tabarrini et al.^96^ (in the blue box), and Lüscher et al.^97^ (in the pink box) along with the IC_50_.

The first potent and selective PARP10 inhibitor is OUL35 (**3**) (Figure 3), which was identified in 2016 by Lehtiö et al.^93^ The authors developed an activity-based assay that was exploited to screen a library of 2638 compounds from the open chemical repository of the National Cancer Institute (NCI) against PARP10. The 19 hits identified were then tested in a dose–response assay, and a few of them showed nanomolar inhibitory activity with compound **3** emerging as one of the most interesting, exerting the desired inhibitory effect without showing significant cytotoxicity against HeLa cells. With an IC_50_ of 330 nM (Table 2), compound **3** was 13–300-fold selective for PARP10 over the other 12 mono- and poly-ARTs tested. Differential scanning fluorimetry (DSF) confirmed the inability of the compound to bind the inactive PARP9 and PARP13, while it stabilized the catalytic domain of PARP10. However, **3** also inhibited PARP14 and PARP15, although at lower potency (IC_50_ = 23 and 4.2 μM, respectively). Compound **3** inhibited PARP10 by interacting with its nicotinamide binding site through the benzamide moiety, as confirmed in DSF by the loss of the activity against a PARP10 inactive mutant in which the NI site is not accessible. Docking studies using PARP10 indicated that **3** would made hydrogen bonds with Gly888 and Ser927 and π-stacking with Tyr919 and Tyr932. In contrast to known PARPi, it extended toward the acceptor site, making hydrophobic interactions with Ile987 and Leu926. The crystal structure of **3** bound to the nicotinamide pocket of PARP15 mutant, gain-of-binding surrogate, confirmed its binding mode (Figure 3).

**Table 2:** Selectivity Profile of OUL35 (**3**) and Its Analogues against a Panel of ARTs (μM IC_50s_) and Activity in the Cell-Based Assay (μM)

	OUL35 (**3**)^a^	**8**^b^	**9**^b^	**10**^c^	**11**^c^	**12**^d^	**15**^d^	**16**^d^	**17**^d^	**18**^e^
PARP10	0.330	0.230	2.4	3.64	0.480	0.290	0.140	0.150	0.160	0.400
PARP15	>10^f^	3.3	1.2	11.0	1.7	2.4	0.400	0.400	0.370	n.t.^g^
PARP14	23	10	0.630	>100	41	≫10	≫10	≫10	≫10	5.2
TNKS1	>100	>100	n.t.	>100	21	>100	>100	>100	>100	n.t.
TNKS2	>100	>100	>100	>100	6.5	>100	>100	>100	>100	n.t.
PARP1	>100	>100	n.t.	>100	>100	>100	>100	>100	>100	>100
PARP2	>100	>100	>100	27	1.7	38	>100	>100	>100	n.t.
cell-based assay^h^	1.35	2	n.t.	1.7	1.8	0.59	1.5	1.6	0.95	n.t.

a Reference (93).

b Reference (94).

c Reference (97).

d Reference (96).

e Reference (95).

f In PARP15 gain of binding mutant (Y576L), IC_50_ = 0.295 μM.

g n.t. = not tested.

h Ability of the compounds to rescue HeLa cells from PARP10-induced cell death.

Compound **3** was also able to enter HeLa cells and rescued the cells in a clonogenic assay from induced PARP10 overexpression in a dose-dependent manner with an IC_50_ of 1.35 μM, while the inactive analogs did not have this effect. The assay is based on the observation that overexpression of wild-type PARP10 but not the catalytically inactive mutant leads to cell death.^98^ Finally, in the presence of 5 μM of **3**, HeLa cells were sensitized to hydroxyurea-induced DNA damage in accordance to the data reported for PARP10 knockdown. The potent and selective PARP10 inhibition replicated also in cells coupled with an unconventional binding mode extending toward the acceptor site made **3** a valid starting point for successive SAR investigations.^94,95,97^

In a follow-up study performed by Lehtiö, Tabarrini et al. in 2018, two series of 34 analogues (mainly phenoxy and benzyloxy derivatives) were purchased and synthesized maintaining one benzamide moiety of hit **3** while exploring the ether linker as well as the second benzamide portion (Figure 3).^94^ When the second amide was replaced by substituents such as the aldehyde (**4**) and carboxylic acid (**5**), good PARP10 inhibition was maintained (710 and 180 nM, respectively). Unfortunately, derivative **5** was completely inactive in a colony formation assay, most probably due to its unsuitable pharmacokinetic properties related to the negative charge. The replacement of one of the two benzamide moieties of **3** with smaller substituents was well tolerated. In particular, a methyl group was sufficient to maintain the activity (**6**, IC_50_ = 2.8 μM), but the presence of the cyclobutyl (**7**) gave nanomolar inhibitory activity (PARP10, IC_50_ = 720 nM) (Figure 3). From the linker exploration it emerged that the oxygen can be fruitfully replaced by a more flexible −OCH_2_. This linker did not tolerate a C-4′-substituted aromatic moiety, but other positions could be functionalized with the *o*-F benzene derivative **8** emerging as the best with an IC_50_ of 230 nM (Figure 3). Compound **8** was profiled against a panel of 13 additional PARP/TNKS enzymes, emerging as highly selective (from 1.5- to >435-fold selective), and the activity was also maintained in cells (IC_50_ = 2 μM). Also, the *m*-bromo substituent was suitable with **9** emerging as a weak PARP10 inhibitor (IC_50_ = 2.2 μM) but endowed with better PARP14 and PARP15 inhibitory activity, as reported in a successive paper (IC_50_ of 630 nM and 1.2 μM, respectively).^99^ Without any PARP2 and TNKS2 inhibition, the compound stood out as selective mono-ARTs inhibitor.

Two additional SAR studies on **3** were published successively (Figure 3, Table 2). In 2021, Lüscher, Lehtiö et al. reported 32 close analogues where the diphenyl ether core was variously decorated in the para and/or meta positions of both rings.^97^ The compounds were initially evaluated for their ability to inhibit PARP10 automodification, and then, the active compounds were tested in a colony formation assay in HeLa cells.^98^ Few compounds emerged as active, and the two best derivatives were the 4-phenoxybenzamide bearing a *p-*cyano group in the second ring, compound **10**, and the 3-phenoxybenzamide **11** (Figure 3), a strict positional analogue of **3**, also reported by Schuler and Ferraris in 2018 (see below).^95^ While compounds **10** and **11** inhibited PARP10 with IC_50_ values of 3.64 μM and 480 nM, respectively, when tested in a cellular context a comparable PARP10 inhibition was detected at values of 1.7 and 1.8 μM. This is likely due to differences on the uptake or the stability of the compounds. They were then tested against a panel of 10 other enzymes, showing some selectivity toward PARP10, followed by PARP15 and PARP2 inhibition in the low micromolar range. Worthy of note is that while the compounds inhibited PARP2, the enzyme with a highly similar catalytic domain, PARP1, was not inhibited up to 100 μM.

On the basis of the good profile of benzyloxy derivative **8**, Tabarrini, Lehtiö et al. in 2022 prepared a series of 15 *p*-methoxy benzamide analogues by mainly using cycloalkyls as a C-4 substituent.^96^ With the aim to lock the amide through intramolecular hydrogen bonds and extend the compounds along the NAD^+^ binding cleft, substituents of different sizes were also placed at the C-2 position. When tested against PARP10, most of the compounds showed inhibitory activity in the submicromolar range with the best compound represented by the cyclobutyl derivative **12** (IC_50_ = 290 nM) followed by cyclopentyl derivative **13** (IC_50_ = 593 nM) (Figure 3). Compound **12** maintained very good activity also in HeLa cells with an IC_50_ of 590 nM, better than those reported for hit compounds **3** and **8**, without showing any significant toxicity. None of the synthesized compounds inhibited the poly-ARTs PARP2 and TNKS2 as well as the mono-ART PARP14. On the contrary, most of the PARP10 inhibitors also showed activity against PARP15 even if at 2–9-fold higher IC_50_ values. In the same work, with the aim to rigidify the amide covalently, a series of 2,3-dihydrophthalazine-1,4-dione derivatives **15**–**17**, variously functionalized at the C-6 position, were prepared. Many of the compounds inhibited PARP10 in the nanomolar range with IC_50_s ranging from 130 to 160 nM, thus emerging as the most potent inhibitors reported to date for PARP10. Their profiling against a panel of PARPs highlighted that the inhibitory activity also extended toward PARP15 with nanomolar activity (IC_50_s from 370 to 400 nM), making them dual PARP10/15 inhibitors. 2,3-Dihydrophthalazine-1,4-diones maintained the PARP10 inhibitory activity also in HeLa cells with IC_50_s from 0.95 to 1.6 μM (Table 2).

Compound **3** was also used by Schüler and colleagues in a paper published in 2018 aimed at identifying PARP10 and/or PARP14 inhibitors (see PARP14).^95^ In this work, the amide diarylether scaffold of **3** was decorated at the C-3 or C-4 position with bulky substituents such as 4′-arylpiperidines/piperazines, previously discovered by the same authors as suitable to impart selective PARP14 inhibition.^100^ Only 1 out of 21 compounds, the 3-phenylpiperidine **18** (Figure 3), emerged as a PARP10-selective inhibitor with an IC_50_ of 400 nM with 15-fold selectivity over PARP14 and without any PARP1 inhibition at 100 μM concentration.

**Figure 4:**
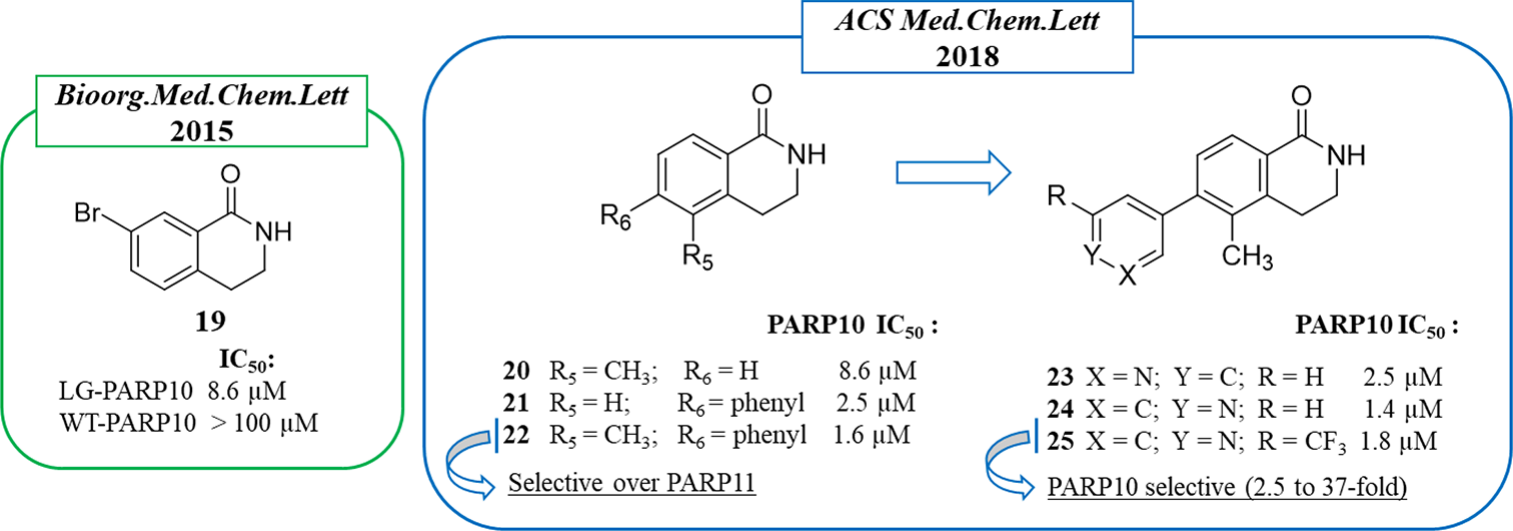
PARP10 3,4-dihydroisoquinolin-1(2*H*)-one (dq)-based inhibitors identified by Cohen et al. in 2015^102^ (in the green box) and 2018^101^ (in the blue box) along with the IC_50_ values against PARP10.

In 2018, Cohen et al.^101^ synthesized a series of 24 3,4-dihydroisoquinolin-1(2*H*)-one (dq) derivatives with the aim of extending the compounds toward a hydrophobic pocket characterizing PARP10 and composed by Ile987 and amino acids Tyr914, Val913, and Ala911 of the D-loop that is less conserved among the various PARP subfamilies. The work moved from a previous study^102^ where the authors applied the chemical genetic strategy of the “bump hole”. In the bump hole approach, the target protein is engineered by removing a bulky residue and thus creating a unique hole where proper substituents can interact. Thus, they prepared two PARP10 mutants, LA-PARP10 and LG-PARP10, against which compounds based on the dq nucleus, already known for its ability to inhibit PARP1,^43,103^ were tested. 7-Bromo derivative **19** (Figure 4) showed the best activity, inhibiting LG-PARP10 with an IC_50_ of 8.6 μM, while no activity against PARP10 wild type and PARP1 was observed up 100 μM. Starting from compound **19**, the C-5 and C-6 positions were explored by introducing different aromatic and nonaromatic substituents (Figure 4). The 5-methyl group emerged as the best, as in compound **20** (PARP10, IC_50_ 8.6 μM), while bulkier substituents, such as the phenyl group, were well tolerated at the C-6 position, as in compound **21** (PARP10, IC_50_ 2.5 μM). By merging the best C-5 and C-6 substituents, disubstituted derivatives were prepared, giving compound **22** that inhibited PARP10 at 1.6 μM with 17-fold selectivity over the other mono-ART tested, PARP11. In order to overcome some aqueous solubility issues, the phenyl ring was replaced by heterocycles, including pyridine, quinolone, 1*H*-indole, and 1*H*-pyrrolo[2,3-*b*]pyridine; the pyridin-3-yl and pyridin-4-yl derivatives (**23** and **24**) showed a similar profile of **22** in terms of both potency and PARP10/PARP11 selectivity. A further structural investigation led to compound **25**, characterized by a 2-CF_3_ pyridin-4-yl at the C-6 position, which maintained an IC_50_ of 1.8 μM against PARP10 and showed a selectivity from 2.5-fold over PARP16 to ≫37-fold over the other 12 ARTs tested. Compound **25** inhibited in a dose-dependent manner both auto-MARylation of PARP10 and MARylation of endogenous PARP10 proteins in HeLa cells.

**Figure 5:**
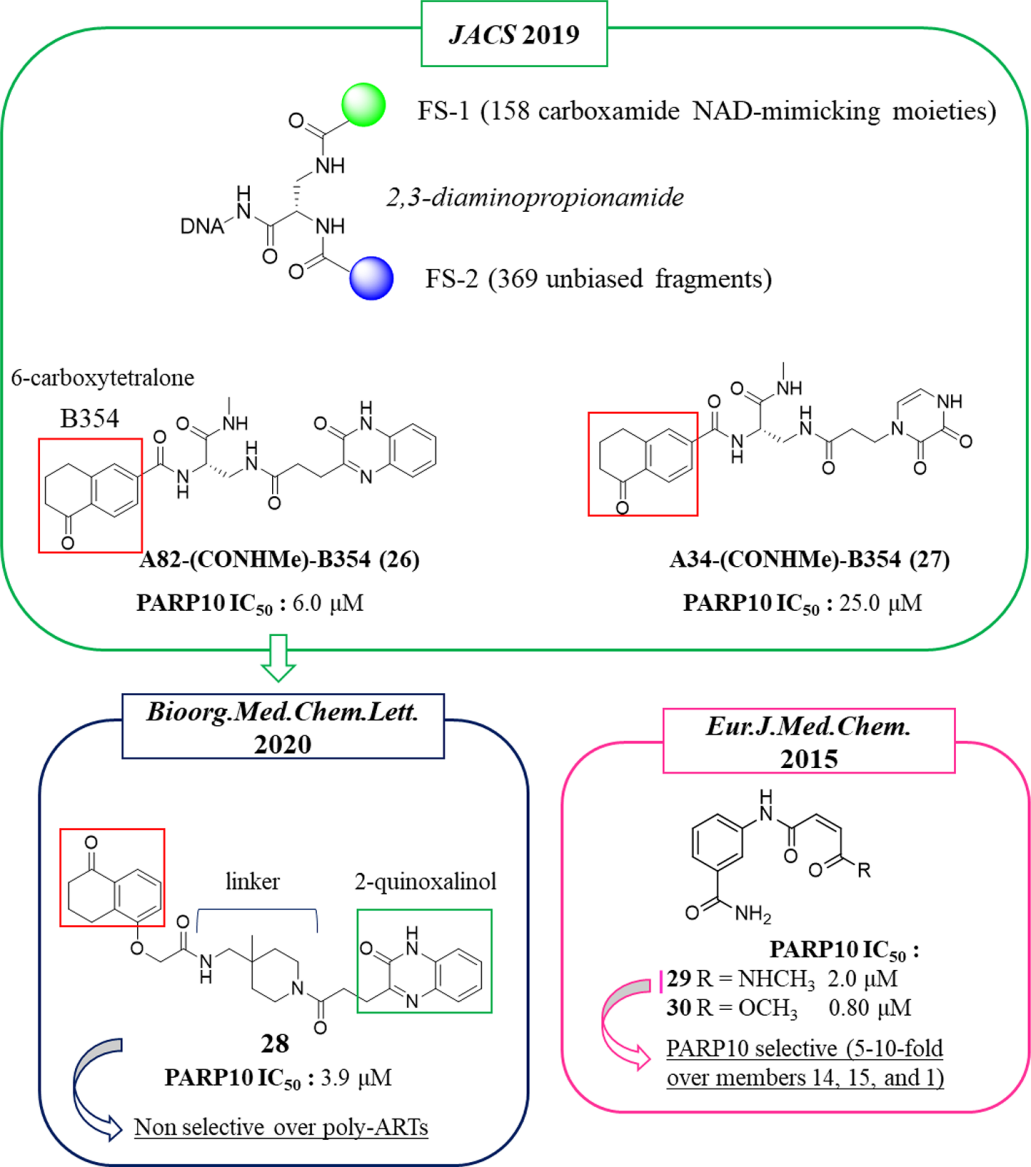
General structure of the compounds belonging to the NAD^+^-mimicking DECL, and structures of the two 6-carboxytetralone derivatives initially identified (in the green box) and its derivative (in the blue box) reported by Franzini et al.^104,105^ 6-Carboxytetralone fragment B354 is underlined with a red box. Structure of 3-aminobenzamide derivatives identified by Schuler et al.^106^ (in the pink box) along with the IC_50_ values against PARP10.

In 2019, Franzini et al. applied the DNA-encoded chemical library (DECL) approach^104^ to identify NAD^+^-dependent enzyme inhibitors. This approach was initially validated to identify PARP1 inhibitors. A 2,3-diaminopropionamide core was functionalized by two fragment sets, FS1 and FS2 (Figure 5). The 158 FS1, which mimicked the carboxamide group of NAD^+^ or other groups usually present in known inhibitors, were combined with 369 unbiased FS2 to give 58 302 DNA-tagged compounds. Overall, the compounds mimicked the NAD^+^ shape, and for this reason they could interact with NAD^+^-dependent enzymes (not only PARPs but also sirtuins or oxidoreductases). By screening the DECL against various NAD^+^-dependent enzymes, disparate compounds emerged that bind mainly the mono-ARTs PARP10, PARP12, or PARP15. Thus, the DNA-free compounds were synthesized and tested against the emerged PARPs. In particular, for PARP10, the well-known benzamide group was identified as a NI mimicking moiety, validating the approach. In addition, the 6-carboxytetralone fragment B354 (red box, Figure 5), never seen before within PARP10 inhibitors, was also identified. This fragment characterized the two inhibitors A82(CONHMe)-B354 (**26**) and A34(CONHMe)-B354 (**27**) that showed IC_50s_ of 6.0 and 25.0 μM, respectively (Figure 5).

Starting from the hit compound **26**, in 2020^105^ 10 different tetralones were virtually coupled with three distinct 2-quinoxalinols through 350 diamine linkers, used to increase drug-like properties, resulting in 10 500 virtual compounds. The designed derivatives were screened in silico using the PARP10 crystal structure. On the basis of the best glide score, chemical feasibility, and diversified linkers, 10 different compounds were synthesized and tested against PARP10. Compound **28**, characterized by a 4-(aminomethyl)-4-(methyl)piperidine linker, emerged with the best glide score (−12.20) and activity against PARP10 with an IC_50_ of 3.9 μM (Figure 5). When evaluated against the other mono-ARTs, PARP3–4, -6, -8–12, -14, and -15, the compound maintained good selectivity for PARP10. It however also showed 100% inhibition of poly-ART PARP2 at 10 μM.

Two additional PARP10 inhibitors, **29** and **30**, were identified by Schüler and colleagues in 2015^106^ while searching for PARP14 inhibitors (see PARP14 for details). The compounds were based on the 3-aminobenzamide nucleus bearing a cis-double bond in the side chain and differ for a methyl amide (**29**) or an ester (**30**) as the terminal moiety (Figure 5). This small structural modification made compound **29** slightly less active (IC_50_ = 2.0 μM) on PARP10 with respect to compound **30** (IC_50_ = 0.80 μM) but markedly more selective over PARP14, PARP15, and PARP1.

### PARP14 Inhibitors for Multiple Cancers

PARP14 (BAL2) is a mono-ART constituted by three ADP-ribosyl binding macrodomains, likely iso-ADP-ribose binding, a WWE domain and a catalytic domain.^107^ The typical catalytic glutamate of PARP1 in PARP14 is replaced by a smaller hydrophobic residue, Leu1782. PARP14 is overexpressed in a series of cancers such as diffuse large B cell lymphoma (DLBCL),^108^ multiple myeloma,^109^ prostate cancer,^110^ and hepatocellular carcinoma (HCC).^111^ The multiple roles of PARP14 in cancer have been recently reviewed.^112^ In cancer cell lines, the overexpressed PARP14 promotes the transcription of genes involved in the growth and cancer proliferation due to its modulation of the IL-4-STAT6 signaling pathway. Indeed, in the absence of IL-4, PARP14 binds the gene promoter and silences the transcription. On the contrary, in the presence of IL-4, STAT6 is activated, and this also promotes PARP14 activation, which in turn dissociates from promoters and enables gene transcription. In this context, PARP14 MARylates histone deacetylases 2 and 3 (HDAC2 and HDAC3) and successively itself, facilitating the activation of transcription cofactors. Another less explored mechanism that correlates PARP14 to the cancer disease is the JNK2–PARP14–JNK1 axis, which seems to be pivotal for malignant multiple myeloma progression. While JNK2 is related to a protective effect of multiple myeloma, JNK1 promotes its apoptosis. Recently, it emerged that in 80% of these tumors, JNK2 regulates the overexpression of PARP14. In turn, overexpressed PARP14 binds JNK1 through its C-terminal domain, thus preventing JNK1-dependent apoptosis.^109,112^ Furthermore, PARP14 emerged as an important effector of the Warburg effect in the HCC. Indeed, PARP14 blocked JNK1-dependent phosphorylation of pyruvate kinase M2 in HCC cells, leading to an aerobic glycolysis promotion. In the absence of PARP14, instead, PKM2 is phosphorylated by JNK1, glucose is converted into pyruvate, and apoptotic processes are enhanced.^111^ Besides the cancer disease, PARP14 dysregulation is also related to other pathological states such as allergic inflammation or atherosclerosis.^113^

**Figure 6:**
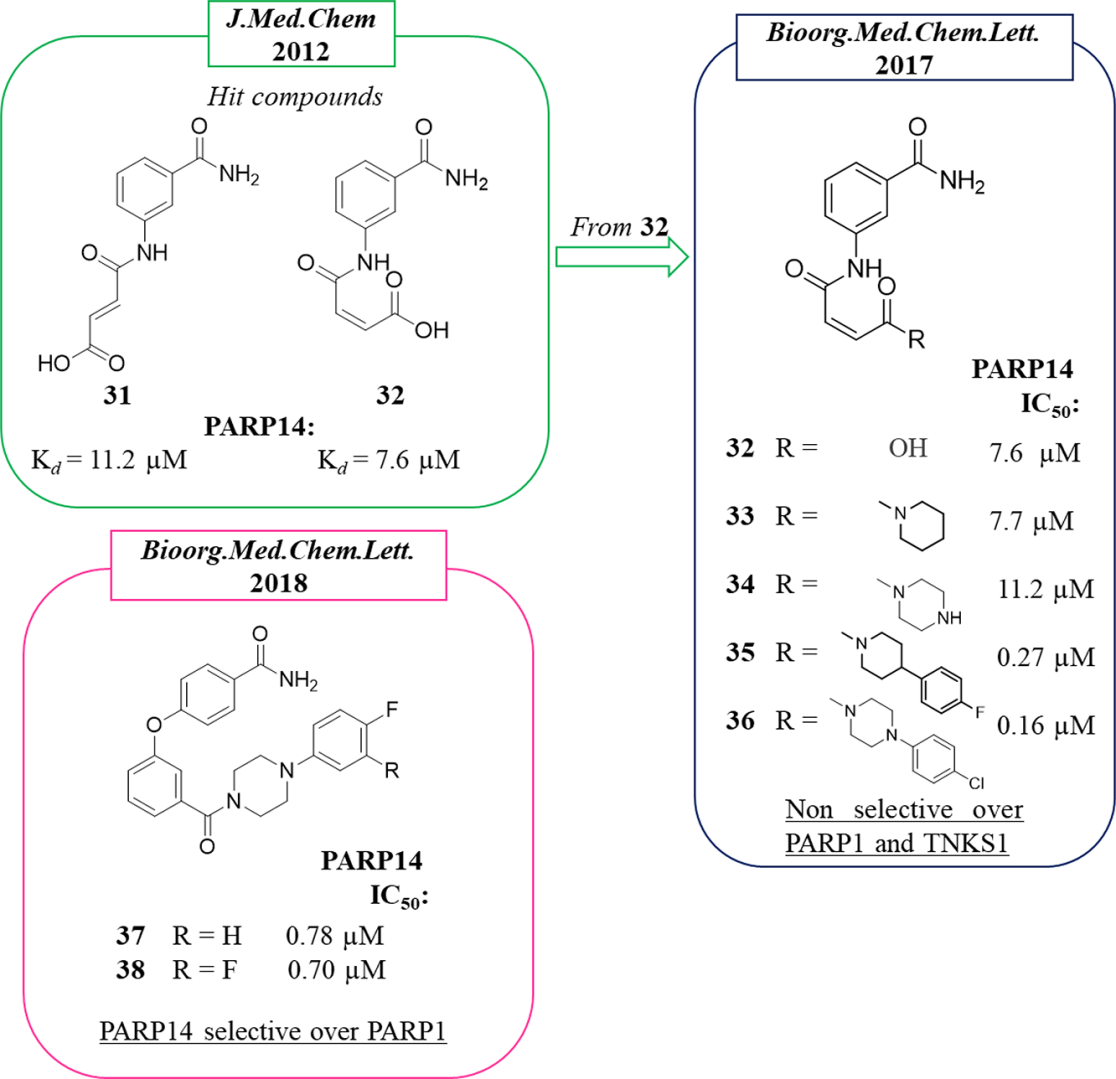
Structures of 3-aminobenzamides reported by Schüler, Linusson et al.^114^ (in the green box) and by Ferraris et al.^100^ (in the blue box) and of diaryl ether compounds reported by Ferraris et al.^95^ (in the pink box) along with the IC_50_ values against PARP14.

Among the mono-ART enzymes, PARP14 is one of the most studied also in the context of inhibitor design. In 2012 Schüler, Linusson et al.^114^ performed a structure-based virtual screening of 8050 compounds in order to identify PARP14 and PARP15 inhibitors. Among the 111 compounds that emerged as initial hits, 14 stabilized PARP14 and 2 stabilized PARP15 in DSF with 4 of them showing selectivity for PARP14 (not 15) over PARP1. On the basis of the docking pose, the authors suggested that the high number of compounds that interacted with PARP14 in comparison to PARP15 could be due by the unique position of Tyr576 in PARP15 that partially constricted the NAD^+^ binding site. The active compounds belong to different classes, but all of them showed the benzamide moiety or its bioisoster triazole. Derivative **31** (Figure 6) showed high selectivity for PARP14 as measured in DSF assay (Δ*T*_m_ > 3 °C) over PARP15 (Δ*T*_m_ < 1 °C) and also over PARP1 (Δ*T*_m_ < 0.5 °C). This compound was based on the classical 3-aminobenzamide nucleus in which the amino group was decorated with a side chain having a double bond with an (*E*) configuration ending with a carboxylic acid. The (*Z*) isomer, compound **32**, was also synthesized to be studied in parallel (Figure 6).

**Figure 7:**
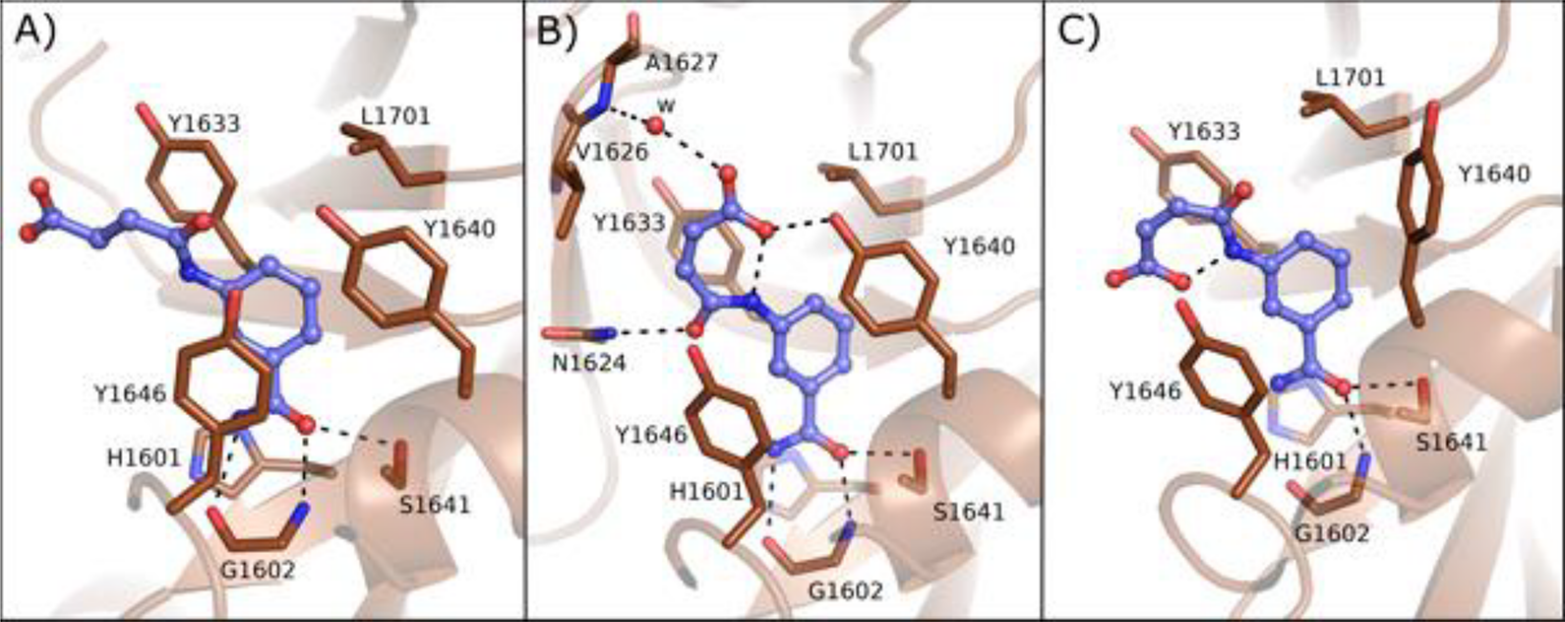
Binding modes of compounds **31** (A) and **32** (B, conformer 1; C, conformer 2) bound to PARP14 (PDB IDs 4F1Q and 4F1L). Residues are numbered according to isoform 1 of PARP14 (UniProt Q460N5-1), and this numbering has been used in all of the PARP14 crystal structures presented in the review.

Through isothermal titration calorimetry (ITC) measurement it emerged that compounds **31** and **32** bound PARP14 with *k*_d_ = 11.2 and 7.6 μM, respectively. They were then cocrystallized with PARP14 with a resolution of 1.9 and 2.8 Å, respectively (Figure 7). From the structures, it emerged that both isomers interacted with the NAD^+^ binding site extending toward the D-loop. **31** showed one binding mode (Figure 7A), whereas **32** showed two binding modes (Figure 7B and 7C) with different conformations, both having an intramolecular interaction between the carboxylic moiety and the amino group. Two tyrosine residues (1633 and 1646) were involved in stacking interactions, and Gly1602 formed typical hydrogen bonds with the amide of conformer 1 that is also involved in an interaction with the hydroxyl group of Ser1641. Conformer 1 interacted also through two hydrogen bonds: the amide carbonyl with Asn1624 of the D-loop and the carboxylic acid with Tyr1640. Furthermore, the acid also had a water-mediated interaction with Val1626 (Figure 7B). However, based on our interpretation, the amide of conformer 2 is too far (3.6 Å) and therefore not able to create a hydrogen bond to Gly1602 (Figure 7C). The selectivity of compound **32** could be explained based on the binding mode of the compounds that showed an interaction with the less conserved part of the NAD^+^ binding site. Indeed, Tyr1640, involved in the interaction, is replaced by Lys903 in PARP1; another difference is represented by Leu1701 that is instead replaced by Glu988 in PARP1.^114^

When compounds **31** and **32** were assayed enzymatically, contrasting results emerged. In 2015, they were reported as inactive in PARP14 with IC_50_ > 20 μM while showing some activity on PARP1, -10, and -15. A successive paper reported for compound **32** an IC_50_ of 7.6 μM on PARP14.^106^ In the same paper, aiming at extending the compound from the NI site to the less conserved ADE subsite or the adjacent D-loop, it was elaborated giving the piperidine derivative **33** and piperazine analogue **34**, which emerged as active against PARP14 with IC_50_ values of 7.7 and 11 μM, respectively, even if they were deprived of selectivity over PARP1 (Figure 6). Some analogues of both piperidine and piperazine derivatives were synthesized that improved the compounds. The activity increased in both series when a bulky substituent was placed at the 4 position of the heterocyclic rings such as the *p*-chlorophenyl in the piperazinyl derivative **36**, which emerged as the most potent for PARP14 but with only 5-fold selectivity over TNKS1 and unselective for PARP1. A better selectivity profile was shown by *p*-fluorophenylpiperidine **35**, which showed an IC_50_ of 0.27 μM against PARP14, 30-fold selective over TNKS1 even if it was still active against PARP1 at the same range. Starting from compound **32** and replacing the carboxylic acid with a simple methyl amide moiety, the PARP10 inhibitor **29** was obtained, as previously mentioned (Figure 3).

Schüler, Ferraris et al. continued to work on these compounds by designing derivatives aimed to discern the SAR against the two related mono-ARTs, PARP14 and PARP10.^95^ To this aim, their 3-aminobenzamide PARP14 inhibitors were merged with PARP10 inhibitor **3**(93) (Figure 3). Among the 21 hybridized compounds, all characterized by a benzamide bearing a *p*-phenyl ether, variously functionalized at the 3 or 4 position, the phenylpiperidine derivative **18** emerged as selective for PARP10, as previously mentioned (Figure 3). On the other hand, when the piperidine was replaced by a piperazine, as in compound **37** (Figure 6), the activity was shifted from PARP10 (IC_50_ of 1.4 μM) to PARP14 (IC_50_ of 0.78 μM) without inhibiting PARP1. The fluorine analogue **38** showed a similar profile (Figure 6). The authors also tested the metabolic stability of **18** and **37** to evaluate the potential of compounds based on the *p*-phenyl ether benzamide. The piperazine **37** showed better metabolic stability in mouse liver microsomes than the piperidine analogue **18**, which was almost completely metabolized after 2 h (85%).

**Figure 8:**
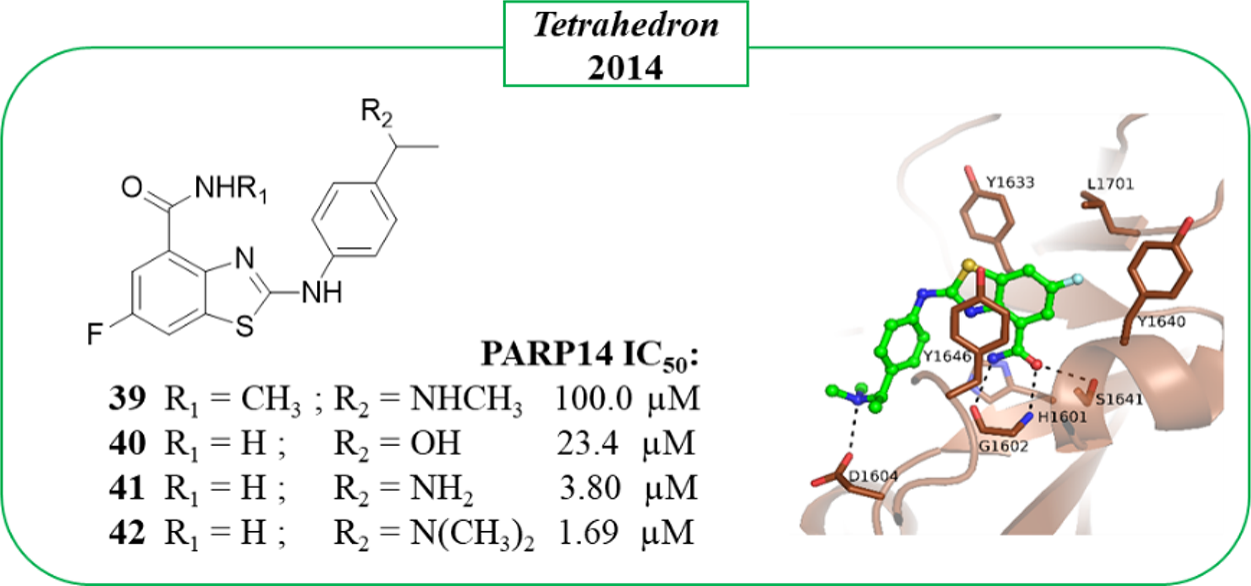
2-Aminobenzothiazole-4-carboxamides synthesized by Zhang et al.^115^ along with PARP14 IC_50_ values; crystal structure of compound **42** in complex with PARP14 catalytic domain as observed in monomer B in the asymmetric unit (PDB ID 4PY4). It is important to note that the amide group of **42** was flipped because it was incorrect in the deposited model and resulted in clashes, and shown in the figure is the corrected model. Error in the original model also pushed the compound out from the very poor density observed in monomer A that was used by the authors to analyze the interaction.

In 2014, Zhang et al.^115^ developed an efficient Pd-catalyzed reaction for the direct coupling of 2-aminobenzothiazole with aryl halide for obtaining 2-arylaminobenzothiazoles. Exploiting this synthetic procedure, a small library of 30 derivatives was prepared, and 4 of them were evaluated for their inhibitory activity in ELISA against PARP14. The compounds are functionalized with a C-6 fluorine atom and C-4 amide coupled with different aminoaryl groups at the C-2 position (Figure 8). With the exception of **39** characterized by a methylated amide, the other derivatives, **40**–**42**, showed good inhibitory activity with the *N*,*N*-dimethylamino derivative **42** emerging as the best (IC_50_ = 1.69 μM, Figure 8). Crystallographic studies confirmed that compound **42** interacted with the NAD^+^ binding site. However, the authors deposited a model where there are clear errors in the compound as well as poor electron density to support the binding mode. We therefore describe here our interpretation of the binding mode (Figure 8). The compound binds to the NI site as other PARP inhibitors and forms typical hydrogen bonds to the glycine and serine residues. It extends along the donor site, and the authors noted that the *N*,*N*-dimethylamino group formed a hydrogen bond with Asp1604. Although compound **42** showed very interesting activity against PARP14, the authors did not explore its selectivity profile.

**Figure 9:**
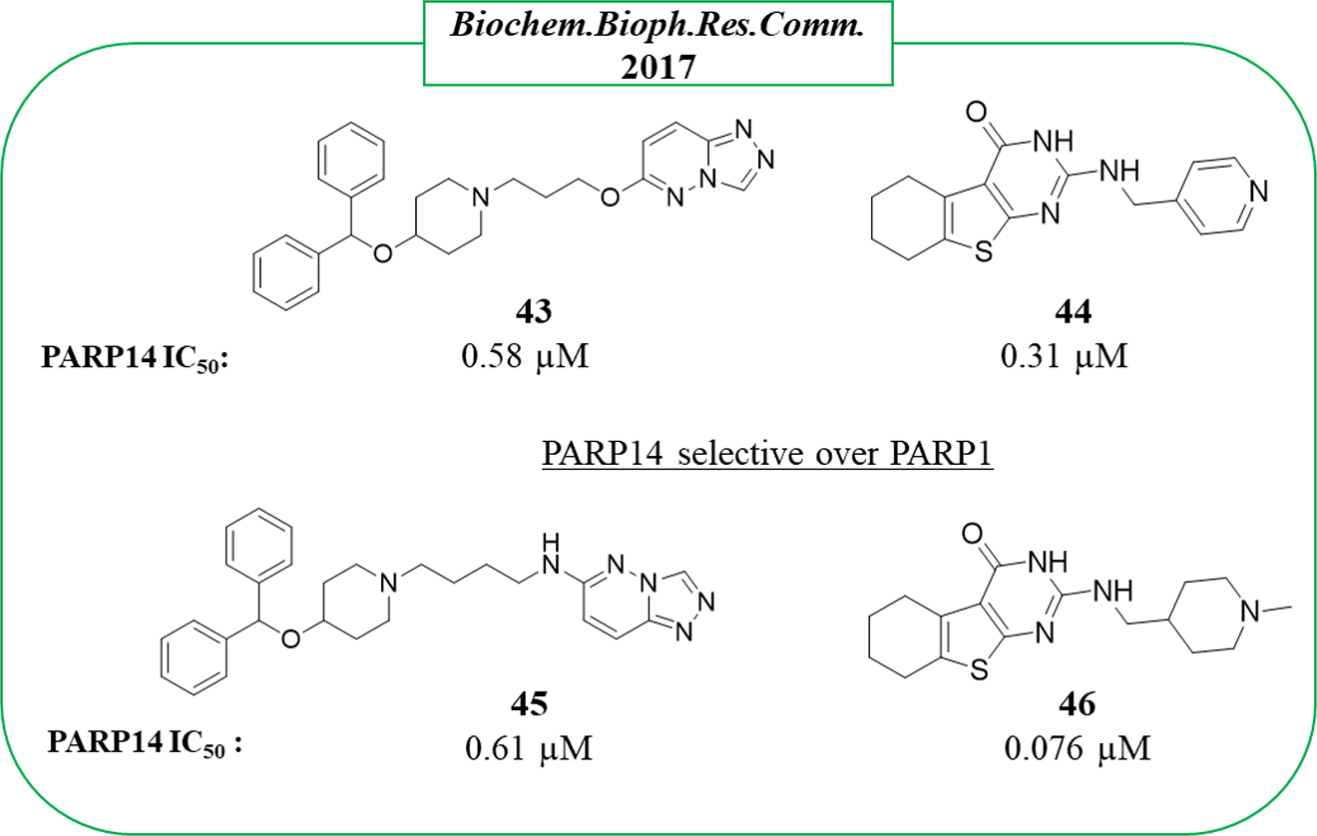
Structures and PARP14 IC_50_s of triazolo[4,3-*b*]pyridazine **43**, benzothieno[2,3-*d*]pyrimidin-4-one **44**, and their derivatives **45** and **46** reported by Yoneyama-Hirozane et al.^116^ along with the IC_50_ values against PARP14.

By screening a library of 500 000 small compounds from Takeda Pharmaceutical Co. using a RapidFire high-throughput mass spectrometry method (which calculated the nicotinamide released from NAD^+^), novel PARP14 inhibitors were identified by Yoneyama-Hirozane in 2017.^116^ Compounds **43** based on a triazolo[4,3-*b*]pyridazine and **44** based on 5,6,7,8-tetrahydro[1]benzothieno[2,3-*d*]pyrimidin-4-one (Figure 9) inhibited PARP14 without significant activity against PARP1 (IC_50_ > 26 μM). The ADP-ribosylation measured by immunoradiometric assay confirmed a submicromolar activity with IC_50_ = 0.58 and 0.31 μM, for **43** and **44**, respectively. Two strict analogues, **45** and **46** (Figure 9), were also tested as active with compound **46** showing the best activity (IC_50_ = 76 nM). Unfortunately, the selectivity for these new compounds was not studied.

**Figure 10:**
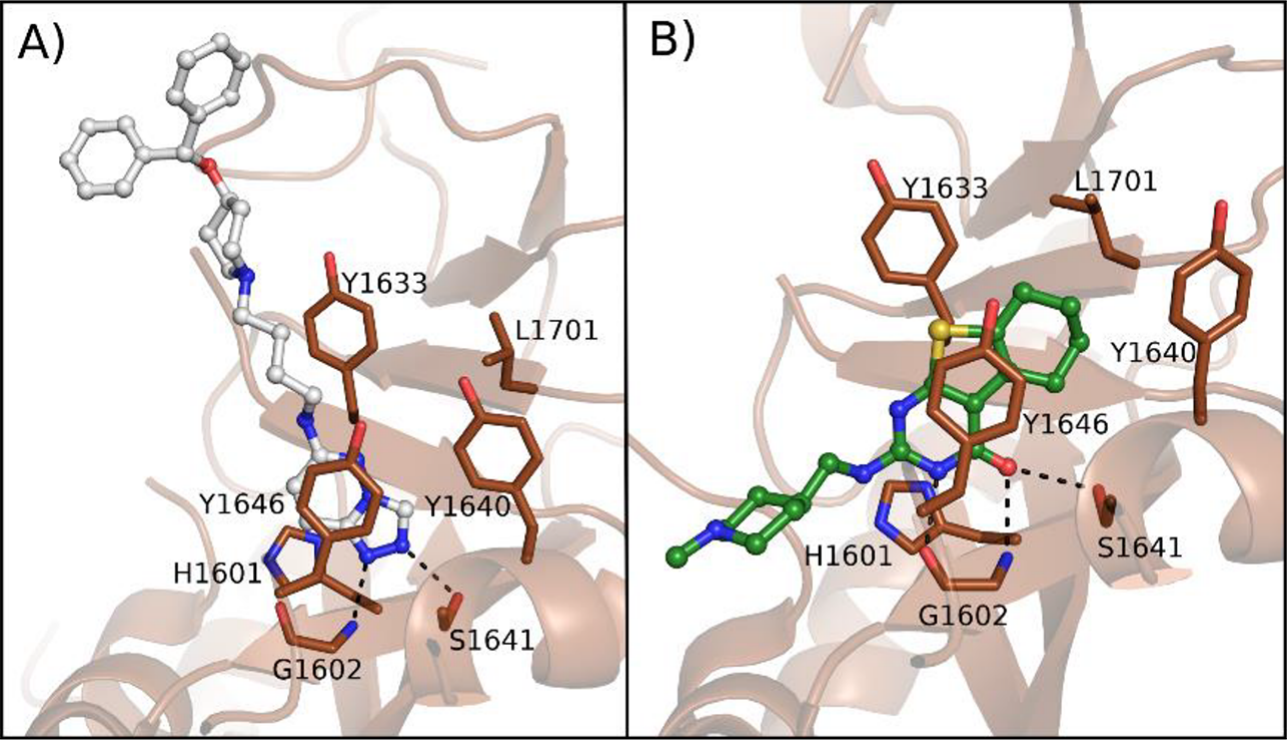
(A) Compound **45** (PDB ID 5V7T) and (B) compound **46** (PDB ID 5V7W) in complex with PARP14.^116^

From their structures with PARP14 it emerged that the compounds interacted with the catalytic domain (Figure 10). Compound **45** made two key hydrogen bonds between two of the three nitrogen atoms of triazole and Ser1641 and Gly1602 residues (Figure 10A). The aliphatic chain and two terminal phenyl rings extend to solvent. Compound **46** formed hydrogen bonds with the same residues of Ser1641 and Gly1602 through the carbonyl group. Gly1602 was also involved in another hydrogen bond with pyrimidine nitrogen (Figure 10B). The terminal methylpiperidine extends the compound toward the ADE site. A comparison with the PARP1 structure showed that the compounds would clash with the residues of the autoinhibitory regulatory domain only present in PARP1–4, and this could explain the reduced activity against PARP1. Compounds **43**–**46** were also cell permeable and recognized intracellular PARP14, as monitored in the intracellular PARP14 stability assay performed in A549 lung cells by accumulation of NanoLuciferase-fused PARP14 (PARP14–NanoLuc) with pEC_50_ values showing correlation with the pIC_50_ values measured with the enzymatic assay. The studies revealed the triazole ring as a suitable replacer of the classic benzamide as a nicotinamide mimic.

**Figure 11:**
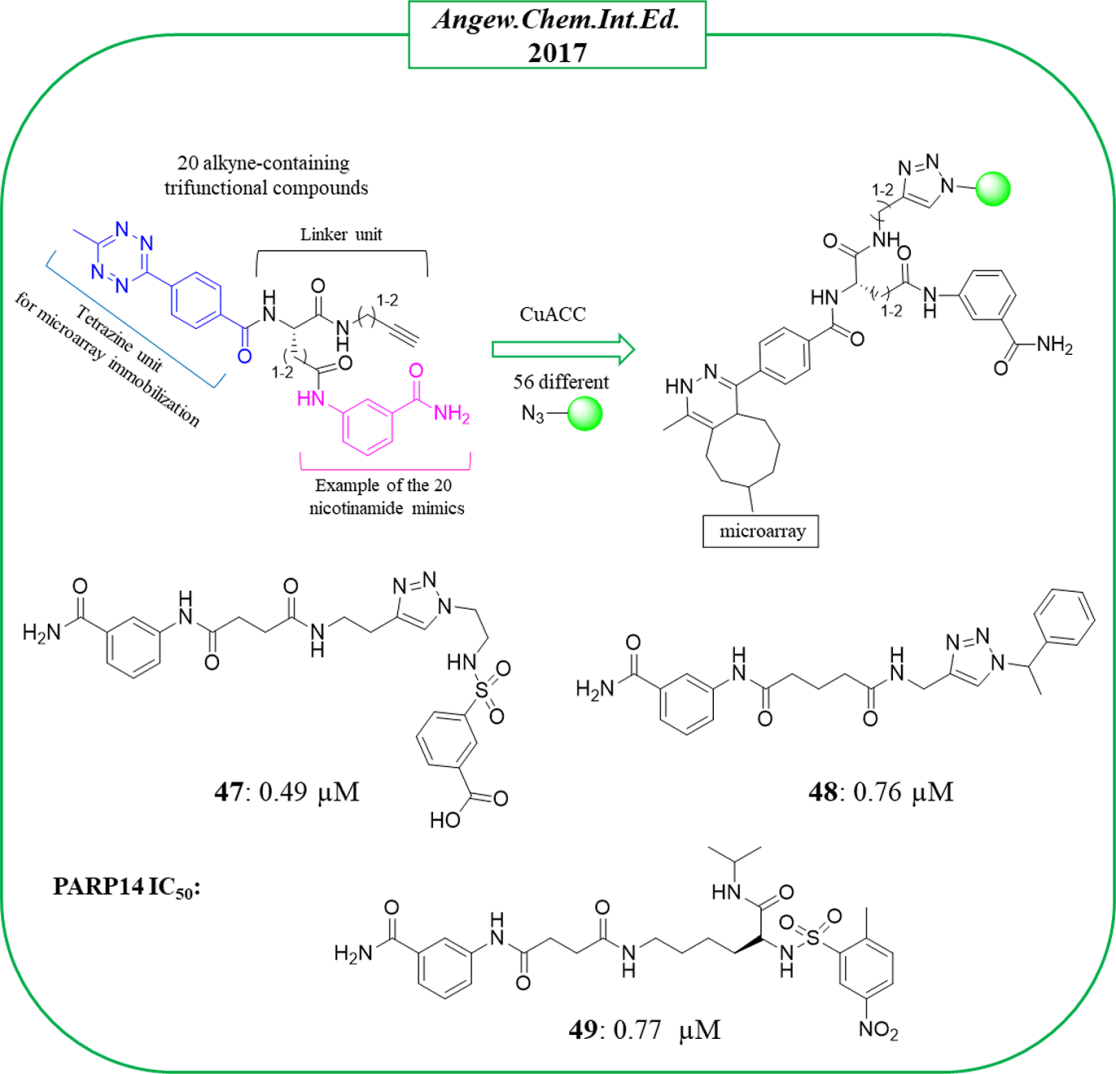
Bidentate PARP14 inhibitors identified by Yao et al.^117^ along with the IC_50_ values against PARP14.

**Figure 12:**
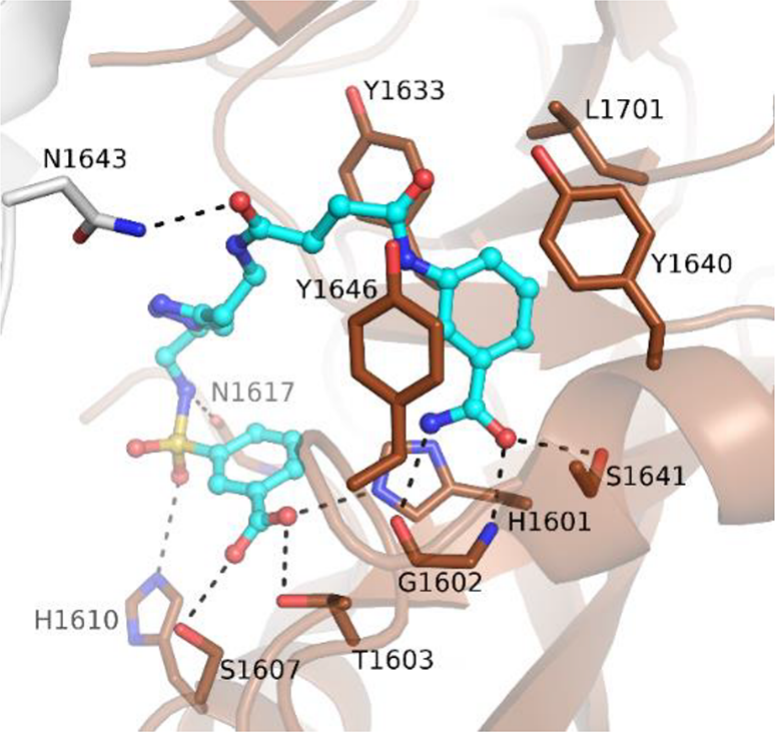
Binding of compound **47** to the PARP14 active site (PDB ID 5LYH).^117^ Asn1643 from a neighbor molecule is colored in gray.

In the same year, bidentate PARP14 inhibitors were identified by Schüler, Yao et al.^117^ The compounds, similarly to inhibitors of TNKS or PARP1/2, were designed to extend from the NI binding site to the close site of ADE. There were 1120 compounds generated through an on-chip CuI-catalyzed azide–alkyne cycloaddition (CuAAC) coupling 20 alkyne-containing trifunctional compounds with 56 different azides (Figure 11). The trifunctional compounds were synthesized starting from five different ligands known for their ability to bind the PARP nicotinamide binding pocket and a tetrazine unit useful for the small molecules microarray immobilization. The two portions were connected through four amino acid-like linkers variable in length and flexibility. The 56 different azides included adenine-mimicking compounds derived from known kinase inhibitors. The microarray was performed against four enzymes (PARP10, PARP14, and TNKS1–2), and 20 hits were identified as potential PARP14 inhibitors. Successively, the corresponding tetrazine-free compounds were synthesized and tested in an in vitro enzymatic assay. Compounds **47** and **48** (Figure 11) emerged as the best, inhibiting PARP14 with IC_50_ values of 0.49 and 0.76 μM and with 24- and 6-fold selectivity over PARP1 and 18- and 4-fold over TNKS1, respectively. They were based on the typical 3-aminobenzamide moiety that from the cocrystallographic studies (on **47**) resided in the NI binding site, as expected based on previously described PARPi. The inhibitor extended toward the ADE binding site with the benzenesulfonamide portion (Figure 12). Compound **47** was then subjected to structural modifications of the triazole ring in order to improve the flexibility. Among the 22 new derivatives, compound **49** (Figure 11), with an opened triazole ring, showed a similar activity of the hit compound against PARP14 with IC_50_ = 0.77 μM. Both **47** and **49** showed good cell permeability and hydrolytic stability. They inhibited the endogenous PARP14 from hepatocellular carcinoma cell line (HepG2) at 10 μM, while they did not inhibit PARP1 in the same cells. A synergism with doxorubicine in HepG2 and dexamethasone in multiple myeloma cell line (RMPI-8226) was also reported.

**Figure 13:**
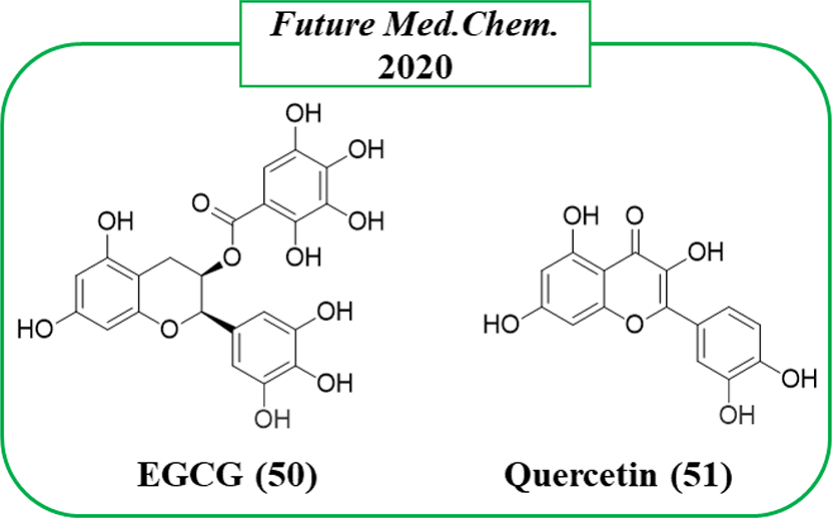
Natural compounds identified as PARP14 inhibitors by Schweiker et al.^118^

In 2020, Schweiker et al.^118^ performed docking studies using the catalytic domain of PARP14 (PDB ID 3SMI) with 60 natural products, already known for various biological activities, which led to the identification of a few virtual compounds. Of them, epigallocatechin-3-gallate (EGCG, **50**), already reported as a mono-ART inhibitor for PARP16,^119^ and quercetin (**51**) (Figure 13) completely inhibited PARP14 at 20 μM with **50** that still maintained the activity at 10 μM. Although these natural compounds could gain attention due to the unusual scaffolds, the lack of a selectivity profile along with the probable promiscuity and possible PAINS behavior could cause alarm regarding their possible progression.^120^

**Figure 14:**
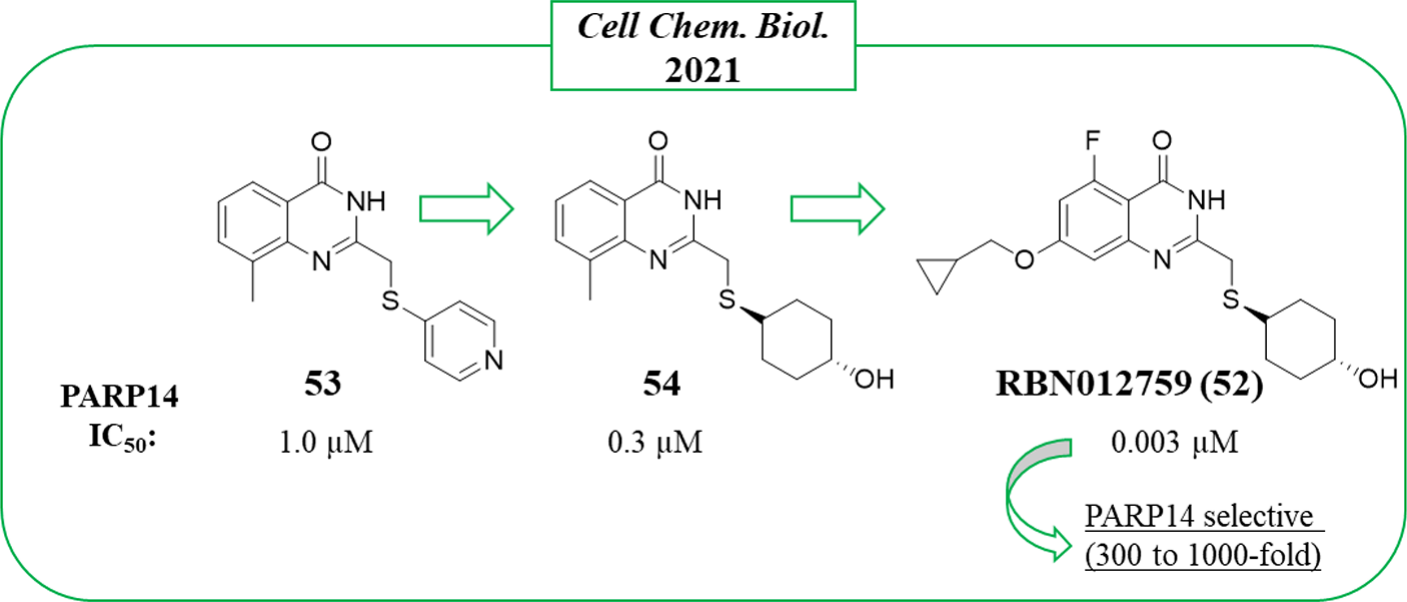
PARP14 inhibitors developed by Ribon Therapeutics^122^ along with the IC_50_ values against PARP14.

In 2021, Ribon Therapeutics published two papers on quinazolinone-based compounds, one reporting the first proteolysis targeting chimera (PROTAC) (see the Alternative Strategies To Inhibit Mono-ARTs section)^121^ and the other that identified the most promising PARP14 inhibitor reported so far, RBN012759 (**52**) (Figure 14).^122^ The study that led to **52**(122) started from compound **53**, which emerged by screening a PARP-targeted compound collection through PARP14 automodification DELFIA (dissociation-enhanced lanthanide fluorescence immunoassay).

**Figure 15:**
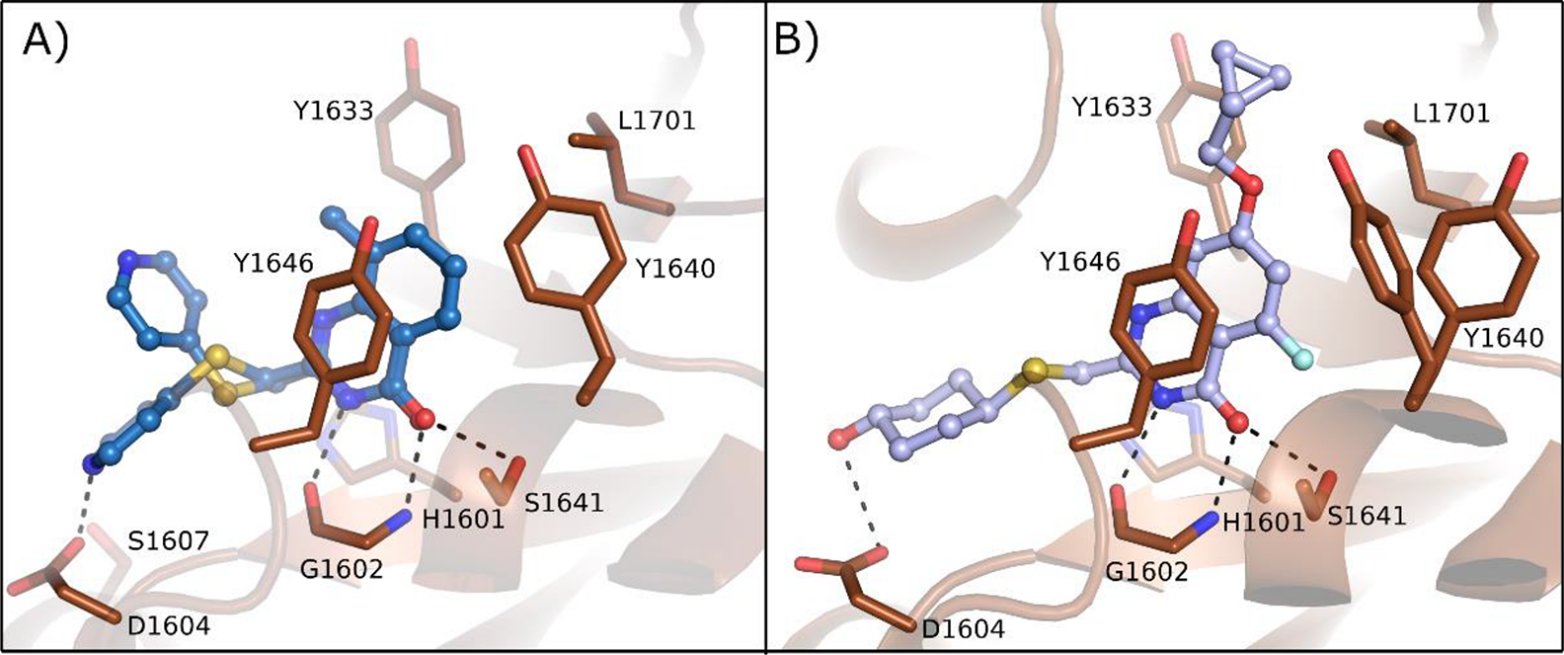
PARP14 crystal structures in complex with (A) **53** and (B) **52**. PDB IDs 6WE4 and 6WE2, respectively.^122^

Compound **53** inhibited PARP14 at 1 μM, but when it was profiled against all of the human PARPs and TNKS enzymes, it emerged as an unspecific inhibitor. Compound **53** bound the NAD^+^ binding pocket (Figure 15A) with the quinazolinone NH that interacted through a hydrogen bond with Gly1602 and the carbonyl with OH of Ser1641; π–π interactions were also performed with Tyr1646. The C-2 thiopyridine substituent instead extended the compound toward the D-loop, very close to Ser1607 and Asp1604, which are unique residues of PARP14 and PARP15. These two residues interacted each other with a hydrogen bond that was hypothesized to be displaced by a proper substituent to achieve selective inhibition. With this aim, the C-2 position was explored with different thioether groups giving 2-*trans*-cyclohexanol **54** (Figure 14) that showed the best inhibitory activity against PARP14 with IC_50_ = 0.3 μM, while it was less active on most of the studied PARPs and above all very selective over PARP15 (IC_50_ = 30 μM) (Figure 14). Additional hydrophobic substituents were successively placed in the benzene ring to reduce the affinity with poly-ARTs that are characterized by a catalytic glutamate, a strategy already applied for the same scaffold.^123^ Compound **52**, having a 7-cyclopropylmethoxy group coupled with a 5-fluorine atom, reached an IC_50_ of 3 nM against PARP14 (Figure 14) with >300-fold selectivity over the other mono-ARTs and >1000-fold selectivity over the poly-ARTs. The cocrystallographic structure (Figure 15B) showed that compound **52** has the same binding mode as **53**. In addition, as planned, the OH interacted with Asp1604, while the C-7 substituent generated a series of van der Waals interactions with the hydrophobic region of PARP14 (Figure 15B). Additional studies showed that compound **52** was endowed with good solubility and permeability properties along with a low MDR1-mediated efflux. It entered the cells and inhibited intracellular PARP14 in a dose-dependent manner with the same IC_50_ value. In addition, compound **52** inhibited PARP14 auto-MARylation in human primary macrophage and in CFPAC-1 (ductal pancreatic adenocarcinoma cells), the latter characterized by a high endogenous level of PARP14, and the PARP14 engagement was confirmed in an in vivo model.^122^

### PARP11 Inhibitors To Reveal Its Cellular Roles

In the catalytic domain an isoleucine residue occupies the third position of the triad. Preceding the catalytic domain PARP11 contains a WWE domain which is involved in the binding of terminal ADP-ribose of poly-ADP-ribosylated proteins.^124^ Very little is known about the physiological and pathological roles of PARP11, but it is involved in the regulation of a nuclear pore complex through the MARylation of some proteins such as Nup98–Nup96.^125^ In 2019, it emerged that MARylation of ubiquitin E3 ligase β-transducin repeat-containing protein (β-TrCP) mediated by PARP11 modulated the activity of type I interferon in the antiviral response.^126^ In particular, mono-ADP-ribosylated β-TrCP promotes IFNα/β receptor subunit 1 (IFNAR1) degradation through a ubiquitination-mediated mechanism. PARP11 is mainly upregulated in response to Zika infections, and by cooperating with PARP12 it suppressed Zika virus replication.^127^

**Figure 16:**
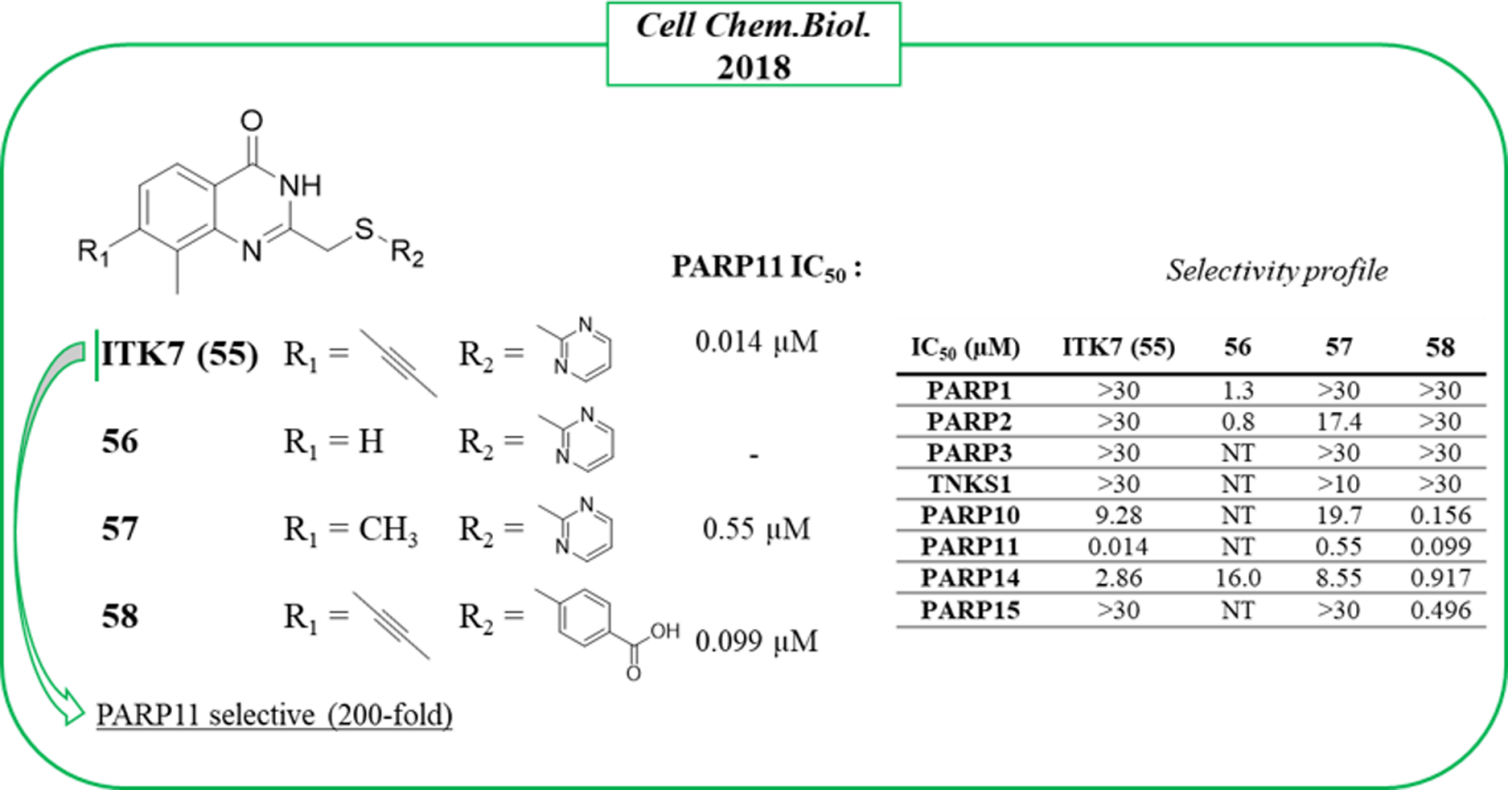
Structural optimization of the quinazolinone scaffold up to the identification of PARP11 inhibitor **55** by Cohen et al.^123^ along with the IC_50_ values against PARP11 and selectivity profile.

ITK7 (**55**) (Figure 16) is the sole potent and selective PARP11 inhibitor reported to date. It was identified by Cohen et al. in 2018,^123^ who explored the quinazolinon-4(3*H*)-one scaffold, the same largely exploited also by Ribon Therapeutics to obtain PARP14 inhibitors, as mentioned above. The study started with compound **56**, which inhibited PARP1 and PARP2 at low micromolar concentrations, and to shift the selectivity in favor of mono-ARTs, small and hydrophobic substituents were placed at the C-7 position. As planned, 7-methylated **57** (Figure 16) did not inhibit any poly-ARTs at concentrations below 10 μM but was active on PARP11 with an IC_50_ of 0.55 μM. A clear preference for mono-ARTs was achieved by inserting a bigger propynyl group coupled with a 2-*p*-benzoic acid, as in compound **58**, which reached PARP11 with nanomolar activity. Selective PARP11 inhibition was then achieved when the propynyl group was coupled with a 2-pyrimidine ring, as in compound **55**, which showed an IC_50_ = 14 nM, 200-fold selective over other five mono-ARTs, and without inhibiting the three poly-ARTs at all. Its selective activity was also maintained in a cellular context with EC_50_ = 13 nM, preventing the auto-MARylation of PARP11 in HeLa cells in a dose-dependent manner without inhibiting PARP1 or PARP10. Given its selectivity and potency, **55** was also exploited as a chemical probe in order to elucidate the enzymatic localization of PARP11 that was identified at the nuclear envelope.^123^

### PARP15 Inhibitors To Reveal Its Cellular Roles

PARP15 (BAL3) contains ADP-ribosyl binding macrodomains at the N-terminus, allowing likely localization and protein–protein interactions enabling the C-terminal catalytic domain to potentially modify the target macromolecules.^107,128^ However, the enzyme has not been studied much, and the cellular roles of PARP15 are still elusive. It is associated with stress granule formation,^129^ and recently, it has been shown that it is able to ADP-ribosylate 5′-phosphorylated ssRNA.^130^ PARP15 is overexpressed in B-aggressive lymphoma,^107^ and there are some implications in the literature that could play a role in acute myeloid leukemia.^131^

**Figure 17:**
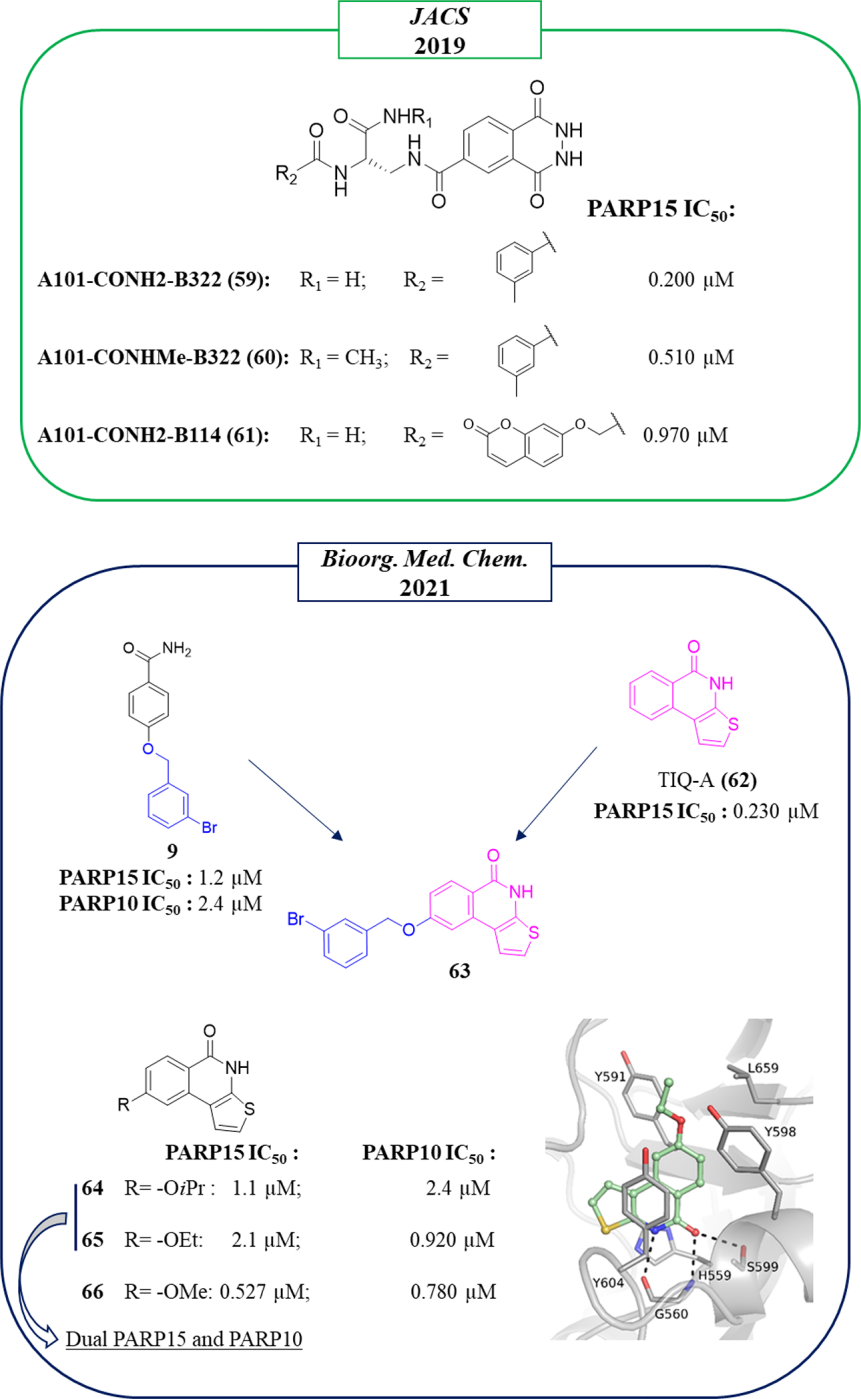
Structures of compounds identified from DECL by Franzini et al. (in the green box)^104^ and of chimera compounds identified by Lehtiö et al. (in the blue box)^99^ along with the IC_50_ values against PARP15. Crystal structure of compound **65** in complex with PARP15 catalytic domain (PDB ID 7OUX).

In the work of 2019 in which Franzini et al.^104^ through the DECL approach identified the already discussed PARP10 inhibitors, anti-PARP15 compounds also emerged (Figure 17). In particular, the 2,3-dihydrophthalazine-1,4-dione fragment was identified as suitable to inhibit PARP15, conferring submicromolar activity to the compounds A101-CONH_2_-B322 (**59**), A101-CONHMe-B322 (**60**), and A101-CONH_2_-B114 (**61**) with IC_50_s of 200, 510, and 970 nM, respectively. Molecular docking studies indicated that compound **59** would bind to the nicotinamide pocket, but the binding mode was not experimentally determined, and the selectivity of the compounds was not evaluated.

The 2,3-dihydrophthalazine-1,4-dione nucleus was confirmed as particularly suitable to inhibit PARP15 also by compounds **15**–**17** (Figure 3). As mentioned before, these compounds were conceived as PARP10 inhibitors, but they actually behave as dual PARP10/PARP15 inhibitors, inhibiting both enzymes with IC_50_s ranging from 150 to 400 nM. The cocrystallographic structures revealed that they work as nicotinamide-mimicking compounds that extend toward the acceptor site and interact with PARP15 through hydrogen bonds with Gly560 and Ser599 and π–π interaction with Tyr604.^96^

Another series of dual PARP10/PARP15 inhibitors was identified by Lehtiö et al,^99^ in a paper aimed at improving the selectivity toward mono-ARTs again by reaching the acceptor site. Through a hybridization approach, TIQ-A (**62**),^132^ known to inhibit PARP1 but also other enzymes in the nanomolar range including PARP15, and the selective mono-ART inhibitor *m*-Br derivative **9** (Figure 17) were merged.^94,133^ Unfortunately, the synthesized chimeric compound **63** (Figure 17) was completely inactive. However, when a small aliphatic substituent was placed at the C-8 position, the thieno[2,3-*c*]isoquinolin-5(4*H*)-ones **64** and **65** emerged as dual PARP15/PARP10 inhibitors in the low micromolar range. The presence of the smallest methoxy substituent gave **66**, the most potent compound on PARP10/PARP15 but at the expense of selectivity, also being active on TNKS2 (IC_50_ = 160 nM). The modest activity of **64** and **65** against TNKS2 is justified by the crystallographic structures that highlight a steric clash between the C-8 substituent and the catalytic Glu1138 of TNKS2 (Leu659 in PARP15, Figure 17).

### PARP6 Inhibitors To Interfere with Mitosis and Cause Cancer Cell Apoptosis

Little is known about the functions of PARP6. The first study aimed at understanding its role was performed by Cohen et al.,^134^ which identified PARP6 as the most relevant mono-ART enzyme involved in the regulation of hippocampal dendrite morphogenesis. Indeed, high levels of PARP6 are present in the brain, particularly in the hippocampus region with an essential role in neurodevelopment from late embryonic to the early postnatal stage. This role also suggested that defective PARP6 could contribute to the pathogenesis of several diseases such as autism and Rett’s syndrome.^134,135^

Successively, identification of the first, even orally bioavailable, PARP6 inhibitor AZ0108 (**67**) by AstraZeneca also shed light on its role in cellular replication.^136^ In particular, it was found that PARP6 MARylates checkpoint kinase 1 (chk1) downregulating its phosphorylation. When PARP6 is inhibited, the chk1 hyperphosphorylation led to the generation of supernumerary centrosomes during mitosis, resulting in a multipolar spindle (MPS), which is toxic for the cancerous cells (mitotic catastrophe). This highlights the important role of PARP6 in mitosis, in particular, in the G_2_-M phase progression.^136^ The PARP6 inhibition or its knockdown in some breast cell lines, such as HCC1806 or MDA-MB-468, induced strong apoptosis that could represent a valid therapeutic approach.^136^

**Figure 18:**
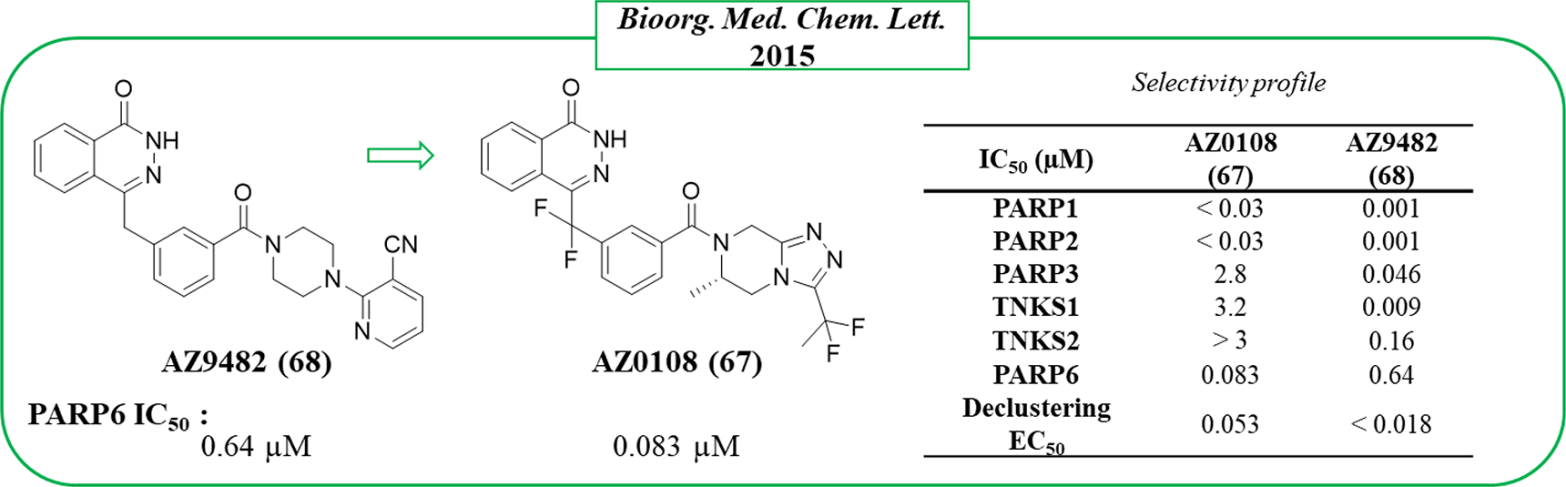
PARP6 inhibitors developed by AstraZeneca (in green box)^137^ along with the IC_50_ values against PARP6, and the table with their selectivity profile.

On the basis of the known role of TNKS2 and PARP16 in the centrosome clustering, in 2015 AstraZeneca assayed their quinazolinone- and phthalazinone-based PARP inhibitors collection in the phenotypic declustering assay using HeLa cells.^137^ This assay evaluates the ability of small molecules to block the centrosome clustering causing MPS. From the screening, compound **68** (Figure 18), based on the same phthalazinone nucleus of olaparib developed by the same company, emerged as a potent inhibitor with an EC_50_ < 18 nM. When the compound was tested against a restricted panel of enzymes (PARP1, PARP2, PARP3, TNKS1, TNKS2, and PARP6), it resulted as a pan inhibitor in the nanomolar range. An optimization campaign led to **67**, which is orally bioavailable with an excellent in vivo pharmacokinetic profile transversally on mouse, rat, and dog. **67** showed a slightly decreased declustering activity (EC_50_ = 53 nM, Figure 18) and a different PARP inhibition profile, becoming much less active on TNKS1, TNKS2, and PARP3 and 6-fold more active on PARP6. Since olaparib tested in parallel showed a very low declustering activity, the authors suggested that the PARP6 inhibition mainly contributed to the declustering of compound **67**. The effect of the PARP6 inhibition by **67** in cell death was evaluated against a series of 18 breast cancer cell lines, active only in HCC1806 and MDA-MB-468.^136^ The activity was confirmed in in vivo xerographic models with more efficacy in the MDA-MB-468 model using a daily oral dosing of 10 mg/kg. Samples from the treated tumors exhibited mitotic defects such as disorganized spindle and MPS, confirming the mechanism of the antitumor activity.

### PARP12 Inhibitors To Effect Cell Stress and Cancers

PARP12 is a member of mono-ARTs containing 5 CCCH zinc fingers, which recognizes viral and cytoplasmic RNAs.^138,139^ It also contains a putative iso-ADP-ribose binding WWE domain preceding the catalytic C-terminal domain which is H–Y–I. The cellular roles of PARP12 are still elusive. Under steady-state conditions, PARP12 is localized in the Golgi complex, while under stress conditions, it translocates to stress granules in a PARP1-dependent manner. Indeed, several PARylated nuclear proteins move to the cytoplasm where they interact with the PARP12 WWE domain, thus promoting the PARP12 accumulation in cytoplasmic stress granules.^129,140^ Herein, PARP12 regulates mRNA translation and stability as a response to a stress conditions.^129^ This translocation is, however, a reversible condition, and after the stress response, PARP12 relocalizes in the Golgi complex.^141^ PARP12 ADP-ribosylates Golgin-97 thereby regulates the basolateral transport of proteins.^142^ The function of PARP12 in cancer remains highly controversial. While a deficiency of PARP12 in HCC was found to be associated with a promotion of migration and invasion of HCC,^143^ in other tumors it seems to be highly upregulated.^144^ In particular, it was recently reported that in MCF7 breast cancer cell lines PARP12 silencing potentiated the effect of the alkylating agent mafosfamide by reducing cell survival and cancer regrowth.^144^ In the latter case, the use of a PARP12 inhibitor could be a good selective strategy to counteract the cancer proliferation.

**Figure 19:**
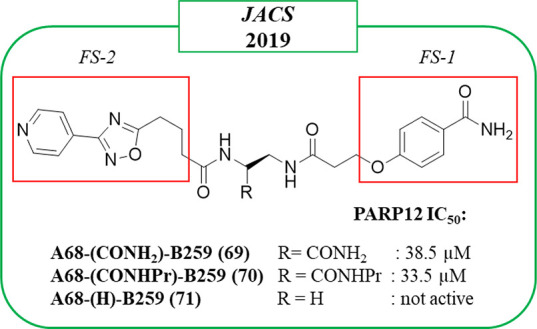
Structures of compounds identified from DECL by Franzini et al.^104^ along with the IC_50_ values against PARP12. Benzamide and 3-(4-pyridinyl)-1,2,4-oxazole fragments are highlighted in red boxes.

Only modest PARP12 inhibitors have been reported that came from the already mentioned work of Franzini et al.^104^ Combination of the benzamide fragment A68 (FS-1, in the red box) and the 3-(4-pyridinyl)-1,2,4-oxazole B259 (FS-2, in the red box) led to A68-(CONH_2_)-B259 (**69**), which showed inhibitory activity against PARP12, IC_50_ of 38 μM (Figure 19). When the CONH_2_ moiety in the linker was replaced with CONHPr, similar activity was displayed by A68-(CONHPr)-B259 (**70**); on the other hand, the presence of a H atom gave the inactive A68-(H)-B259 (**71**) (Figure 19).

### PARP16 Inhibitors To Control Unfolded Protein Response

PARP16 is another member of mono-ARTs in which the catalytic glutamate is replaced by isoleucine. It is the only member that, to date, was demonstrated to be associated with the nuclear envelope and endoplasmic reticulum through a C-terminal transmembrane domain.^145,146^ It plays essential roles in the regulation of unfolded protein response and in response to stress.^7^ Worthy of note, PARP16 deletion is associated with the formation of toxic protein aggregates that could reduce cancer cell growth.^147^

**Figure 20:**
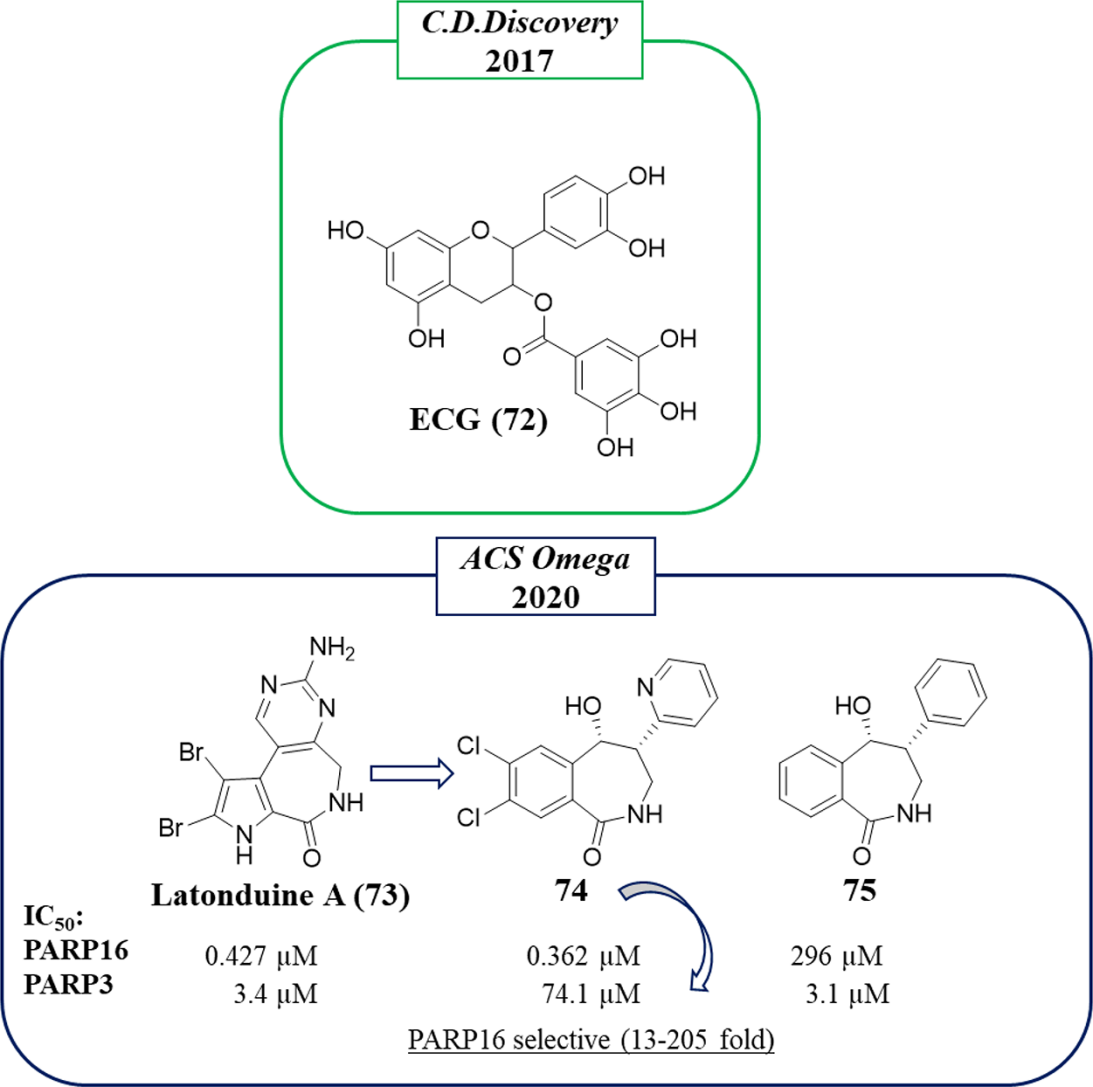
Structures of natural compounds identified by Wu et al. (in the green box)^119^ and of derivatives reported by Andersen et al. (in the blue box)^148^ along with the IC_50_ values against PARP16 and PARP3.

To identify PARP16 inhibitors, in 2017,^119^ 3375 small molecules were screened using a microarray, including natural compounds from traditional Chinese medicine, FDA-approved drugs, and known inhibitors. Nineteen showed high affinity for PARP16, and this activity was confirmed at 0.5 mM by an in vitro auto-ADP-ribosylation assay with the natural compound epicatechin-3-gallate (ECG, **72**) (Figure 20) that completely inhibited the catalytic activity. A binding affinity assay showed for this compound *K*_d_ = 3.41 nM, 2-fold more potent than its strict analogue **50** (Figure 13) assayed in parallel (*K*_d_ = 6.16 nM). The inhibitory activity of PARP16 was then evaluated in vitro, and **50** showed an improved inhibitory activity with an IC_50_ of 14.52 μM compared to **72** that was 47.18 μM. There is however a large discrepancy between the reported dissociation constant and the IC_50_ values.

As the reported potencies in binding assays and in enzymatic assays differ substantially, further studies should be needed to confirm the inhibition mechanisms. Compound **50** was however used as a chemical tool for better understanding the PARP16 role.^119^ To this aim, the relationship between PARP16 activity and two ER stress sensors, PERK and IREα phosphorylation, was investigated. From the data it emerged that PARP16 is essential for PERK phosphorylation, and any PERK activation was instead observed in the presence of **50** as well as in PARP16-deficient QGY-7703 cell lines. In addition, **50** was also responsible for a 10% increase of death in wild-type cell lines, in agreement with the knowledge that a PARP16 deficiency made the cells more susceptible to the ER stress; indeed, no apoptosis was detected in the negative PARP16-deficient cells after treatment with **50**, used as a control.^119^

Other natural products were found to be active against PARPs, such as latonduine A (**73**) (Figure 20), from a marine source, that was identified as a PARP3 and PARP16 inhibitor,^149^ and this inhibition was translated into the ability to correct a F508del-cystic fibrosis transmembrane regulator (CFTR), a chlorine ion channel blocked in cystic fibrosis. In pathologic conditions, chlorine ions cannot flux out of the cells, and this determines the accumulation of thick mucus in the lungs. In order to separate the PARP16 and PARP3 inhibitory properties of **73**, in 2020, Thomas, Andersen et al.^148^ synthesized a library of ∼30 analogues characterized by a simplified structure based on a 2,3,4,5-tetrahydro-*H*-benzo[*c*]azepin-1-one core differently decorated on the two rings (Figure 20). All of the compounds were preliminary tested at 10 μM against PARP3 and PARP16. Two compounds emerged as active with an opposite selectivity profile. Indeed, while compound **74** was 205-fold selective for PARP16 (IC_50_ = 362 nM) over PARP3, compound **75** emerged as selective for PARP3. Profiling compound **74** against a restricted panel of ARTD family enzymes (PARP1, PARP2, TNKS1,2, and PARP4), it emerged to be from 13- to 70-fold selective. Surprisingly, neither compound **74** nor **75** separately showed F508del-CFTR corrector activity, while an activity comparable to **73** was recovered using a combination of the two compounds. Thus, the dual PARP inhibition of **73** justified its effect as a corrector potentially useful to treat cystic fibrosis.

### PARP7 Inhibitors as Anticancer Drugs

PARP7 is a mono-ART also known as tetrachlorodibenzo-*p*-dioxin (TCDD)-inducible poly(ADP-ribose) polymerase (TiPARP). Besides the catalytic domain with H–Y–I, it is characterized by a *N*-terminal nuclear localization signal (NLS), CCCH-type zinc finger domain able to bind RNA, and WWE domain that interacts with iso-ADP-ribose and can mediate protein–protein interactions.^150^ PARP7 is involved in a variety of cellular processes including innate immune regulation, cellular stress response, and transcription factor regulation. Its expression in most of the human tissues is regulated by aryl hydrocarbon receptor (AHR),^151^ liver X receptor (LXR),^152^ and type I interferon (IFN-I).^153^ In 2021, two important papers^154,155^ were published around the PARP7 involvement in cancer, even if they highlighted two opposite PARP7 engagements. Indeed, while Rasmussen et al. demonstrated that PARP7 functions as a tumor suppressor in E2-responsive breast cancer cells,^154^ a PARP7 inhibitor reached clinical trials for its promising antitumor activity.^155^ In the first study, the authors provided evidence that PARP7 is a fundamental member of the negative feedback that regulates estrogen receptor α (ERα). Since ERα represents the main regulator of estrogens in breast tissue and is one of the principal targets for breast cancer treatment, its negative regulation mediated by PARP7 could be beneficial in this kind of cancer.^154^ On the contrary, the downregulation of INF-I response in which PARP7 is involved could be responsible for a reduced T-cell-mediated antitumor activity.^155^ Thus, PARP7 could represent an immunotherapeutic target, and through its inhibition, anticancer activity can be achieved.

Another important role of PARP7 has been recently identified also in ovarian cancer, in which PARP7 contributes to the cancer proliferation. Indeed, PARP7 MARylated various cytoskeletal proteins, including α-tubulin, thus facilitating cell growth; when PARP7 was depleted, a reduction in cancer cell growth is observed.^156^

**Figure 21:**
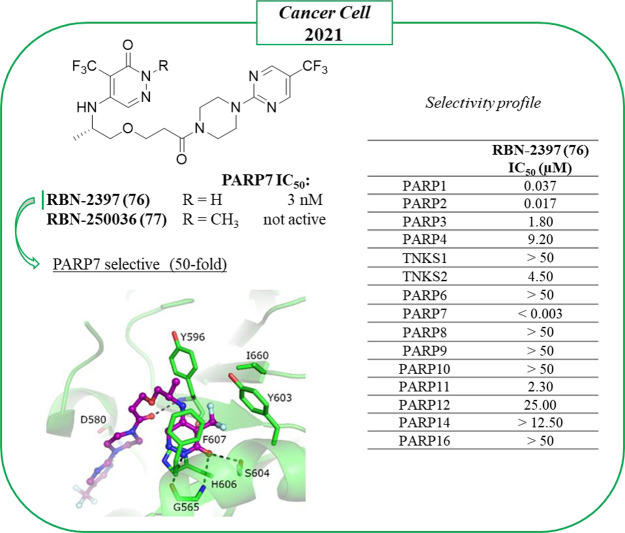
Development of **76** by Ribon Therapeutics, currently under clinical investigation,^155^ along with the IC_50_ values against PARP7, selectivity profile, and crystal structure with a mutated PARP12 mimicking PARP7 (PDB ID 6V3W).

Ribon Therapeutics developed the first PARP7 inhibitor, RBN-2397 (**76**) (Figure 21),^155^ which is based on the pyridazinone nucleus, previously identified by fragment screening as a privileged structure to inhibit mainly mono-ARTs and used to obtain NAD^+^ competitive probes for PARP enzymes.^157^ Compound **76** inhibited PARP7 by interacting with the NAD^+^ binding pocket of the enzyme with an IC_50_ < 3 nM and a *k*_d_ of 0.22 nM. It was >50-fold selective over all of the other catalytically active human PARPs and showed an IC_50_ of 2 nM in SK-MES-1 cells. The PARP7 inhibition mediated by the compound completely abolished the MARylation of TBK1, and this restored the IFN-I signaling. On the contrary, using the inactive methylated analogue RBN-250036 (**77**) (Figure 21), no effect was detected.

The binding mode of **76** was determined with a complex structure of a mutated PARP12 mimicking PARP7. The compound makes five hydrogen bond interactions with residues Gly565, Tyr596, Asp580, and Ser604. In addition, one of the mutated residues, Phe607, creates a π–π interaction with the compound. The antitumor effect of **76** was confirmed using CT26 tumor-bearing mice. Significant tumor growth inhibition was detected at all doses with complete and durable regression after treatment with 30 mg/kg for 6 weeks. Since it was speculated that the PARP7 inhibition led to an immunomodulation responsible for the anticancer activity, also immunodeficient NOG mice with CT26 were treated with **76**, and as expected, no tumor regression was observed. CD8 T cells emerged as being responsible for much of the antitumor immunity induced by the compound. The anticancer effect was also observed in most xenograft models with tumor growth inhibition ranging from 49% to 69% and complete tumor regression in NCI-H1373 lung cancer xenograft. The promising preclinical results led to **76** being investigated in phase I clinical trials that are currently ongoing in patients with advanced or metastatic solid tumors (NCT04053673).^155^

## Alternative Strategies To Inhibit Mono-ARTs

A generally known challenge in PARP inhibitor development is to gain selectivity due to the high sequence similarity among ARTs family NAD^+^ binding pockets. To overcome this issue, alternative ways to mediate and modulate ADP-ribosylation signaling are emerging. A valid approach that can be applied without the difficulties of obtaining inhibitors specific for individual ADP-ribosyltransferase domains could rely on interfering with accessory domains through allosteric modulators.^158^

**Figure 22:**
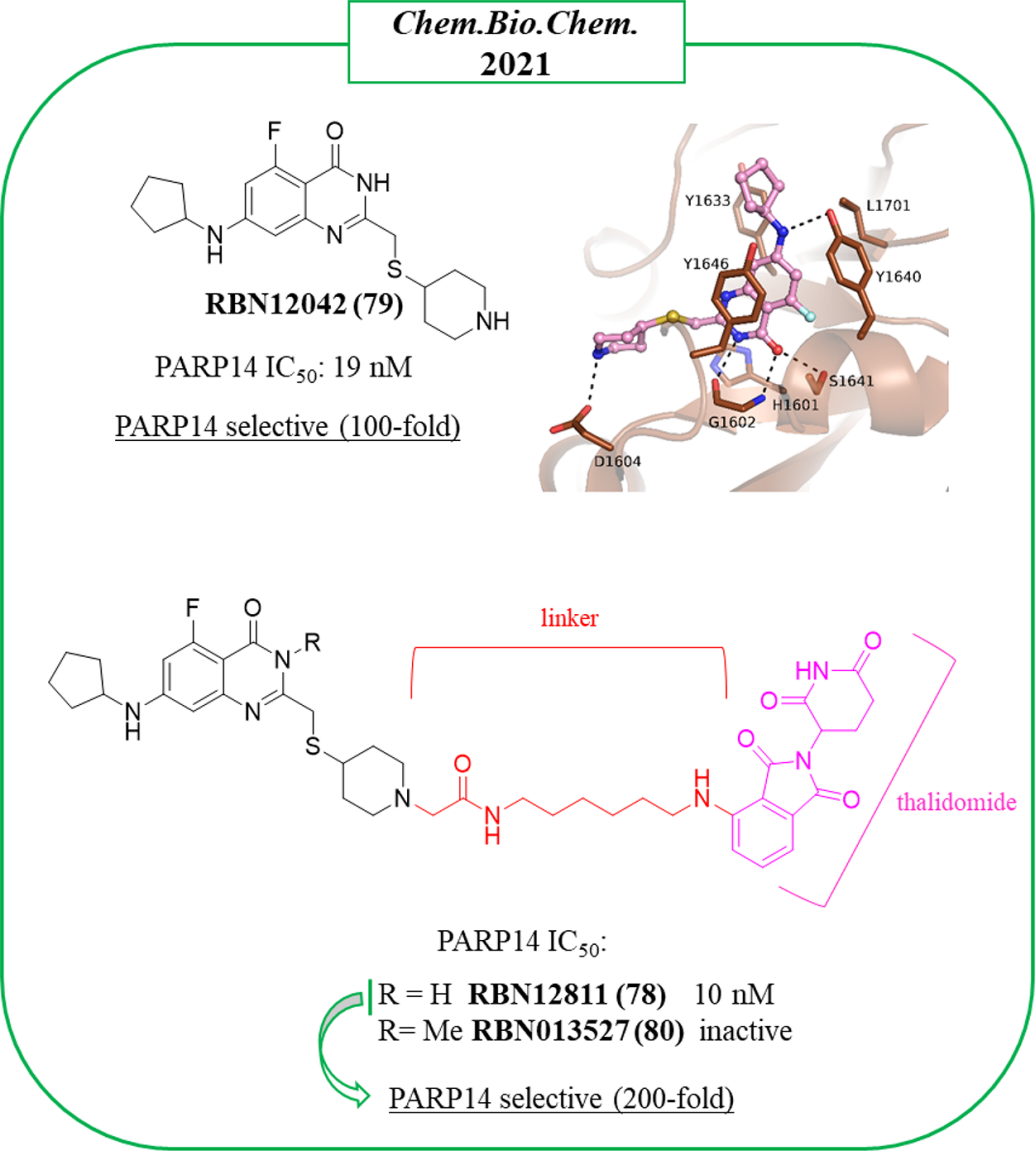
(Top) Compound **79** with its structure in complex with PARP14 (PDB ID 7L9Y) and (bottom) its PROTAC derivative **78**(121) along with the IC_50_ values against PARP14 and their selectivity profile.

An alternative way to interfere with the enzyme would be through reducing the proteins levels with a PROTAC approach. Several PROTACs for poly-ARTs have been already reported as potent and efficacious to treat cancer,^159−161^ while only one degrader, has been developed for mono-ART PARP14 by Ribon Therapeutics,^121^ RBN12811 (**78**). The etherobifunctional degrader **78** (Figure 22) was obtained by tethering the selective PARP14 inhibitor RBN12042 (**79**) to thalidomide through an appropriate linker. Compound **79**, which was developed by the same company, inhibited PARP14 with an IC_50_ of 19 nM, more than 100-fold selective over all of the other human ART enzymes. The 7-NH of **79** makes an interaction with Tyr1640 while Tyr1646 is involved in a stacking interaction with the bicyclic ring. The carboxamide generates hydrogen bonds with Ser1641 and Gly1602. Asp1604 also makes a hydrogen bond with the pyrrolidine group (Figure 22). PROTAC **78** was as active as **79** with an IC_50_ of 10 nM and more than 200-fold selective over all of the other ARTs. The compound was tested in KYSE-270 cells (human esophageal squamous cell carcinoma) where degradation of endogenous PARP14 was detected without affecting the levels of the other ARTs. The degradation was also confirmed in MDA-MB-231 (human breast adenocarcinoma) and JJN-3 (lymphoma cell line) as well as in HEK293T and in human macrophages. The methylated analogue RBN013527 (**80**) was inactive, highlighting the need for the secondary benzamide moiety. Degrader **78** was also tested in the presence of three different proteasome inhibitors, confirming that the compound mediated the destruction of PARP14 via a ubiquitin-proteasome pathway. Thus, **78** represents the founder of mono-ART PROTACs, but recent identification of potent and selective inhibitors of a single family could definitely expand this strategy.

PARPs are known to interact with multiple other macromolecules. Protein–protein interaction (PPI) disruptors could provide alternative ways to inhibit these enzymes. PPIs are critical regulatory events in many biological processes representing a precious source for possible new therapeutics, but PPIs are difficult to target, and compounds binding to these interfaces are not commonly found in HTS because PPI inhibitors and traditional drug-like compounds occupy quite different chemical spaces. While still largely underexplored, a few examples have already been reported as PARP-1 inhibitors able to interact with the BRCT domain.^162^ Efforts are also ongoing to prevent interaction of TNKS with their target protein peptides.^163,164^ PPI inhibition could be a potential new avenue to explore also for mono-ARTs as we know more about their target proteins and regulatory mechanisms.

Mono-ARTs contain multiple accessory domains enabling the enzymes to bind to mono- (macrodomains) and poly-ADP-ribosylated targets (WWE domains), allowing them to possibly carry out further modifications on different residues by their catalytic domains. Small molecules have been discovered in two papers that inhibited the macrodomain 2 (MD2) of PARP14.^165,166^ The inhibition of MDs is very promising to obtain selective inhibition, since these accessory domains characterize only three ARTs (PARP9, -14, and -15).^167^ The inhibitors would interfere with the localization and PPIs while not necessarily affecting the catalytic activity itself.

**Figure 23:**
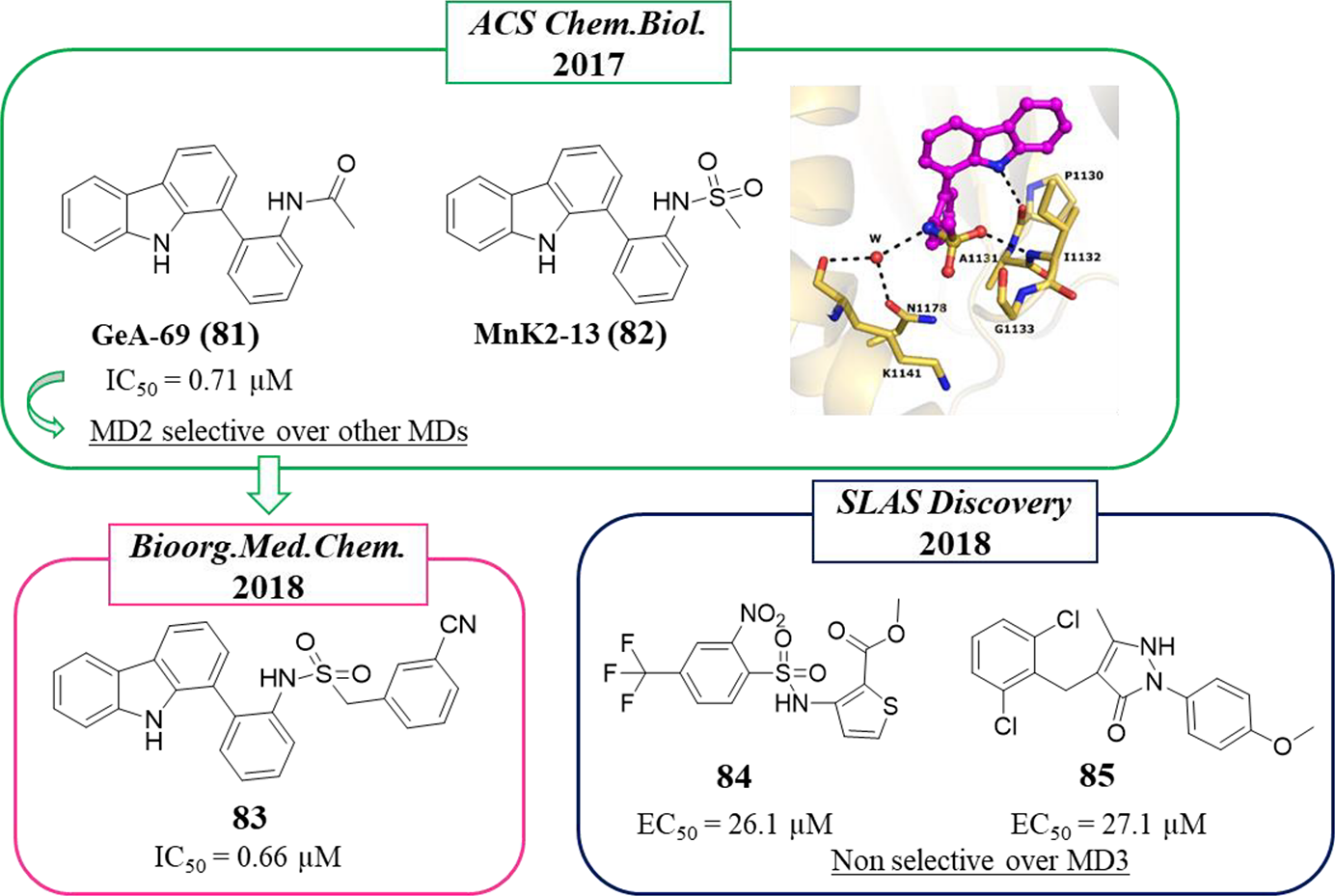
PARP14 macrodomain-2 (MD2) inhibitors developed by Knapp et al. (in the green and pink boxes)^165,168^ and Ekblad et al. (in the blue box)^166^ along with the their inhibitory activity against MD2 and their selectivity. Crystal structure (PDB ID 5O2D) shows the binding mode of **82**.

The first allosteric inhibitor dates back to 2017 when Knapp et al.^165^ described a screening of 48 000 small molecules (3000 in-house kinase inhibitors and 45 000 form Novartis) against MD2 using an AlphaScreen assay that led to the identification of the carbazole derivative GeA-69 (**81**) (Figure 23). This compound, at 80 μM, fully inhibited the binding of ADP-ribose to MD2 with a calculated IC_50_ of 0.71 μM. In order to evaluate the binding mode of the compound, the authors cocrystallized the corresponding methanesulfonamide compound MnK2–13 (**82**) with a PARP14 MD2 mutant. From the structure solved at 1.6 Å, it emerged that it was bound to a hydrophobic site adjacent to the ADP-ribose binding site of MD2 (Figure 23). The interaction determined that a loop from Pro1130 to Pro1140 was pushed into the ADP-ribose site, thereby displacing ADP-ribose. A hydrogen bond was formed between the carbazole NH and the backbone carbonyl of Pro1130 and another between the sulfonamide carbonyl and amino groups of Ile1132. Compound **81** was tested against 11 other MDs, confirming its selectivity in inhibiting only PARP14 MD2. In the presence of a high concentration of **81** (250 μM), the recruitment of PARP14 MD2 to the DNA damage was completely prevented, confirming the inhibitory activity of the compound also in cells (U-2-OS). However, the compound showed some cytotoxicity at about 50 μM against HeLa, U-2-OS, and HEK293T cells after 72 h of incubation. Thirty analogues of **81** were successively synthesized,^168^ highlighting the essential role of the NH of the carbazole and leading to the identification of a new derivative, the 3-cyanophenylmethanesulfoamide carbazole **83** (Figure 23), displaying similar PARP14 MD2 inhibition (IC_50_ = 0.66 μM). Unfortunately, the cytotoxicity was not tested.

In the same year and using again an AlphaScreen method, different PARP14 MD2 small molecule inhibitors were identified by Ekblad et al.^166^ On testing 1584 compounds provided by Chemical Biology Consortium Sweden, 17 compounds were active and were then evaluated by SPR, leading to the identification of the thiophene derivative **84** and the pyrazole derivative **85**, which showed *k*_d_ = 8 and 162 μM, respectively, with respective AlphaScreen EC_50_ values of 26.1 and 27.1 μM, respectively (Figure 23). These compounds also inhibited PARP14 MD3.

## Concluding Remarks and Future Perspectives

The discovery and therapeutic application of PARPi represents a successful approach to targeted cancer treatment due to their excellent ability to selectively kill tumors with deficiency in DNA repair proteins through the so-called synthetic lethality.^38^ After olaparib was introduced to clinical use for the treatment of tumors harboring a defective HRR pathway,^45^ other PARPi reached the market and many other compounds are at various stages of clinical studies, where they are being investigated as a single agent or in combination against diverse cancer types.^52^ PARP1 is generally considered the major target of the FDA-approved PARPi, but due to the structural similarity of the targeted NAD^+^-binding domain, they also inhibit the activity of other PARPs, mainly PARP2. The effect of a polypharmacology in this context is still unknown, and selectivity over the other PARPs may not even be in all cases the most beneficial property of a drug candidate in a clinical setting.^169^ On the contrary, to be useful as a chemical probe for the analysis of the biological response and in proof-of-concept in vivo studies, high specificity of the inhibitor is important. The lack of access to a complete panel of sensitive, high-throughput PARP assays^117,170−172^ to fully evaluate selectivity is a common weakness that leads to profiling of PARP inhibitors only against a handful of family members, rarely including representative mono-ARTs. This also emerged from the present perspective, where only a few research teams profiled their compounds against a larger panel of PARPs and TNKS, but the recent assay development efforts will likely make the profiling against a PARP panel more routine in the future.^173,174^ The lack of selective inhibitors has until now hampered the unveiling of the pathophysiological roles of many mono-ARTs.

The first study that aimed at identifying selective inhibitors of a mono-ART, PARP14, dates back less than 10 years ago.^114^ Since then, 28 papers have been published with an increasing trend during the past few years. In this review, we collected the literature and focused the discussion on those molecules that have been designed or were found to be active against one enzyme or to some extent restricted toward the mono-ARTs class. The SAR studies and hit-to-lead optimization phase have taken advantage of the crystal structures, which in many cases were solved in complex with the inhibitors and the target or a surrogate mono-ART. The selectivity profile against focused or a wide panel of mono- and poly-ARTs was almost always recorded, but the cytotoxicity was tested in parallel only in some cases. Similarly, while the activity against the enzymes was confirmed in cells for many compounds, the pharmacological effects were rarely tested, likely due to the still modest potency and unspecificity of the disclosed early compounds. Most of the small molecules herein reported are indeed still in their infancy as the pharmacokinetic profile has been studied only for the most advanced inhibitors coming from the pharmaceutical companies.

**Figure 24:**
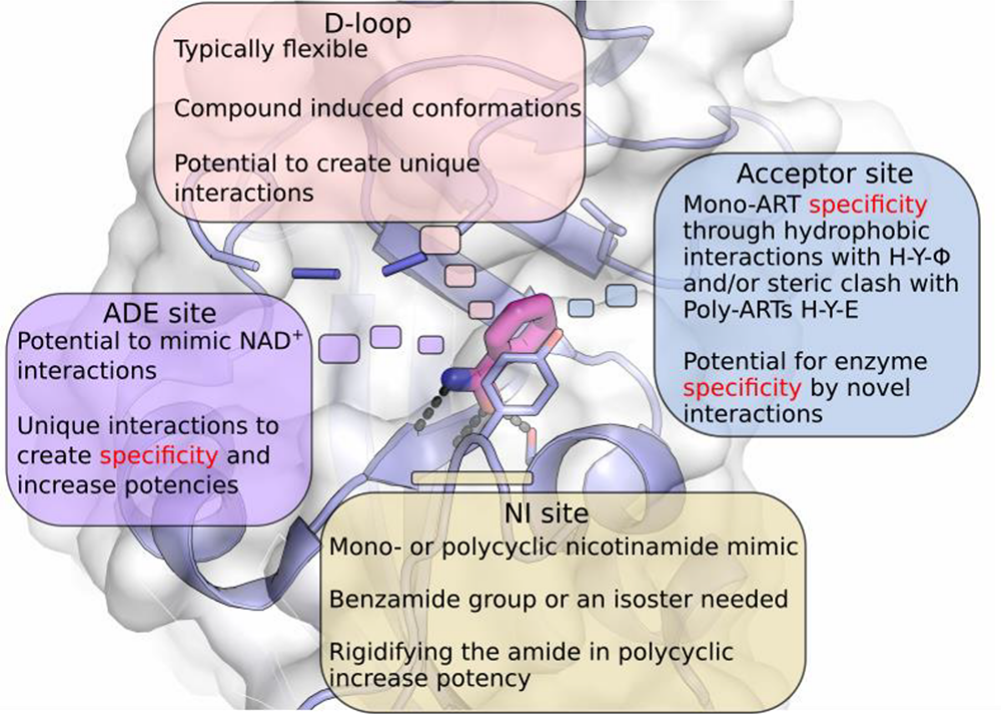
Extended PARP inhibitor pharmacophore highlighting the routes to improve mono-ART inhibitor selectivity while targeting the conserved NI binding site of the catalytic domain.

With the exception of TNKS inhibitors, all of the potent PARPi with defined binding modes share the key features^170,175^ described for the early PARP1 inhibitors. These include the requirements of hydrogen bond donors and acceptors allowing interactions with the backbone amide and carbonyl of a glycine as well as side chain hydroxyl of serine located at the bottom of the NI site. The NI binding pocket is also lined with aromatic tyrosine residues packing against NI of NAD^+^ or the competitive inhibitors. From the efforts described here to discover inhibitors specific toward mono-ARTs, this pharmacophore can be extended to include additional features: some shared with poly-ARTs to increase inhibitor potency and some to gain selectivity toward mono-ARTs through structure-based design (Figure 24).

Analogously to the inhibitors of poly-ARTs, most of the mono-ART inhibitors interact with the NI binding site (Figure 24). Thus, they possess a nicotinamide mimic moiety, usually represented by a bicyclic or polycyclic ring, but a monocycle is also tolerated in mono-ART inhibitors like in OUL35 (**3**) when coupled with the essential primary carboxamide (Figure 24). The carboxamide can be also included in a cycle. This rigidifies the amide by fixing it to a certain conformation and providing entropy gains, a strategy often used in PARPi development. A few structures have emerged as particularly privileged to obtain mono-ART inhibitors such as quinazolinones and phthalazinones that although widespread also among poly-ARTs inhibitors when properly functionalized permitted one to achieve potent and selective RBN012759 (**52**) (PARP14), ITK7 (**55**) (PARP11), and dual compounds **15**–**17** (PARP10/PARP15). In a very few cases, the typical benzamide group is missing, such as in PARP10 tetralone-based inhibitors, **26**–**28**, and PARP14 inhibitors, **43** and **45**, in which the benzamide moiety was replaced by the triazole bioisoster group. The typical feature of mono-ARTs, the “H–Y−Φ” motif replacing H–Y–E of poly-ARTs, provides a way used to restrict the activity toward mono-ARTs due to unfavorable interactions with the negatively charged glutamate present in PARP1–4 and TNKS. PARP10 inhibitor OUL35 **(3**) contains a *p*-ether benzamide and extends toward the acceptor site and toward the hydrophobic Ile987 (Figure 24). Similarly, in the quinazolinone derivatives RBN012759 (**52**) and ITK7 (**55**), the presence of a hydrophobic substituent in C-7 imparted selectivity against PARP14 or PARP11, respectively, by interacting with Leu1782 (PARP14) or Leu1701 (PARP11).

PARPi, like olaparib, extend from the NI site toward the ADE site and span the whole NAD^+^ binding cleft of the ART domain. Analogously to poly-ARTs inhibitor design, also some mono-ART inhibitors extend beyond the NI site, reaching the ADE site. This design would make the compounds larger in terms of MW but would also increase potencies through new interactions and potentially also selectivity by targeting unique residues. As an example, a bidentate compound **47** extends to the ADE site, giving potent and selective PARP14 inhibition. Also, other mono-ART inhibitors were designed to interact with the unique residues in PARP14, such as RBN012759 (**52**), where the *trans*-cyclohexanol interacted with Asp1604, displacing the intra-hydrogen bond with Ser1607 and becoming selective over the other mono-ARTs. Similarly, RBN12042 (**79**) forms interactions with the Asp1604 at the ADE site.

The D-loop lining the NAD^+^ binding cleft is often found to be flexible and disordered in the structures, even when the binding pocket is occupied by a ligand.^76,167^ The possible interactions observed with this region have to be therefore carefully evaluated for using them in inhibitor design. In addition, this region is often involved in crystal contacts with the adjacent protein contributing to the observed conformation (Figure 12). However, these features could also be utilized efficiently in the design of specific inhibitors as shown by compounds **31** and **32**, which inhibited PARP14 (Figure 7) by capturing specific conformation of the D-loop.

There is still a long way to go to study in a more precise and systematic manner the implications of mono-ARTs in the development of cancer as well as in other diseases. Significant advances have been reported, and parallel work of multiple laboratories has elucidated possibilities to create specific inhibitors. Mono-ART inhibitors are now emerging that satisfy Frye’s five criteria for quality chemical probes for interrogating their biological roles in cells and in animal models,^176^ possibly validating these enzymes as drug targets for human diseases.

## Abbreviations Used

ADE site: adenine ribose binding site
AHR: aryl hydrocarbon receptor
AMOT: anigomotin
APC: adenomatous polyposis coli
ARH3: ADP-ribosyl hydrolase 3
ARTD: diphtheria toxin-like ADP ribosyltransferase
CFTR: cystic fibrosis transmembrane regulator
chk1: checkpoint kinase 1
CuAAC: CuI-catalyzed azide–alkyne cycloaddition
DECL: DNA-encoded chemical library
DELFIA: dissociation-enhanced lanthanide fluorescence immunoassay
DLBCL: diffuse large B cell lymphoma
D-loop: donor loop
DSB: double-stranded break
DSF: differential scanning fluorimetry
dq: 3,4-dihydroisoquinolin-1-(2*H*)-one
ECG: epicatechin-3-gallate
EGCG: epigallocatechin-3-gallate
EMA: European Medicines Agency
ERα: estrogen receptor α
FDA: Food and Drug Administration
HCC: hepatocellular carcinoma
HDAC: histone deacetylase
HPF1: histone PARylation factor 1
HRR: homologous recombination repair
IFN-I: type I interferon
ITC: isothermal titration calorimetry
LXR: liver X receptor
MD: macrodomain
MPS: multipolar spindle
NAD^+^: β-nicotinamide adenine dinucleotide
NCI: National Cancer Institute
NI: nicotinamide
NLS: nuclear localization signal
PARP: poly-ADP-ribose polymerase
PARG: poly-ADP-ribose glucohydrolase
PARPis: PARP inhibitors
PCNA: proliferating cell nuclear antigen
PPI: protein–protein interaction
SSB: single-stranded break
SAR: structural–activity relationship
TCDD: tetrachlorodibenzo-*p*-dioxin
TNKS: tankyrase
ZnF: zinc finger
